# A Study on a Sector-Shaped Cantilever Diaphragm for Frequency-Response Improvement of an Acoustic Sensor Based on Piezoresistive Effect in MOSFET

**DOI:** 10.3390/s26196274

**Published:** 2026-10-03

**Authors:** Chayeong Kim, Eunsik Noh, Wonkyu Moon, Kumjae Shin

**Affiliations:** 1 Department of Mechanical Engineering, Pohang University of Science and Technology (POSTECH), Pohang-si 37673, Republic of Korea; kcycj@postech.ac.kr (C.K.); sikhaem@postech.ac.kr (E.N.); 2 Construction Equipment Technology Center, Advanced Mobility & Robot System Group, Korea Institute of Industrial Technology (KITECH), Gyeongsan-si 38408, Republic of Korea

**Keywords:** acoustic sensor, complementary metal oxide semiconductor, field-effect transistor, micro-electromechanical acoustic sensor, micro-electromechanical system, piezoresistive effect, sector-shaped cantilever diaphragm

## Abstract

This study investigates an acoustic sensor that exploits the piezoresistive effect of a field-effect transistor (PrFET) with a sector-shaped cantilever that improves frequency-response characteristics. PrFET energy transduction is capable of detecting acoustic signals by adopting an acoustically responsive sensing structure. However, it has not been investigated whether PrFET acoustic sensors can exhibit signal-response characteristics that differ from those previously reported. To investigate whether the sensor response could be enhanced at a specific frequency, this study adopted a sector-shaped cantilever diaphragm. The sector-shaped cantilever has a free end at the center, allowing for greater stress to be induced in the piezoresistive channel under acoustic excitation. The acousto-mechanical response of the proposed structure was characterized in terms of its displacement under acoustic excitation and the resulting stress induced in the PrFET at the support. A PrFET acoustic sensor incorporating the designed diaphragm was then fabricated, and the sensitivity enhancement and the formation of a specific resonance frequency was experimentally validated.

## 1. Introduction 

Advances in AI analysis technologies require an abundance of acoustic signals. Accordingly, miniaturized acoustic sensors are becoming increasingly important, as they can be applied in large numbers with low power consumption [1]. In addition, if sensors are optimized for target applications, signal acquisition and processing can be more efficient [2]. Some applications, especially, require selective detection at a specific frequency. Manufacturing processes such as laser welding and cold spraying generate sound in the 1~2 kHz range that is closely related to the process conditions [3,4]. Acoustic signals generated by defects in fluid pipelines are also located within the 1~2 kHz [5,6]. Furthermore, in the field of bionomy, population monitoring of endangered amphibians can be conducted by measuring vocalizations within the 1~1.7 kHz [7]. Therefore, the development of acoustic sensors that are sensitive to a specific frequency range will allow efficient signal acquisition.

At a fundamental level, the design of acoustic sensors involves investigating the physical principles that convert acoustic signals into electrical signals. These principles can be divided into mechano-electrical and acousto-mechanical transduction [8]. The electric signal is generated from a mechano-electrical transduction element such as an air-gap capacitors, piezoelectric films or piezoresistors. The mechanical motion or deformation of the elements is converted into electric signals. These elements should exhibit sufficient mechanical excitation from an acoustic input, so acousto-mechanical transduction through an acoustic sensing structure is required. The development of an acoustic sensor with the desired characteristics involves both the selection of a mechano-electrical transduction element and the design of a suitable acoustic sensing structure.

For mechano-electrical transduction, capacitive and piezoelectric mechanisms are the most widely used in commercial miniaturized acoustic sensors [9]. Capacitive sensors employ a diaphragm and a backplate separated by an air gap as the transduction element, whereas piezoelectric sensors employ a piezoelectric film. Piezoresistive acoustic sensors, on the other hand, have been investigated as an alternative. Piezoresistive sensors detect acoustic signals through changes in the resistance of piezoresistors located at stress-concentrated regions of the sensing diaphragm [10,11]. Compared with conventional capacitive and piezoelectric sensors, piezoresistive sensors can offer the following advantages. First, because piezoresistors occupy only localized regions of the diaphragm, their transduction performance is less dependent on the size of the acoustic sensing structure [12]. In capacitive and piezoelectric sensors, by contrast, the transduction performance depends on the capacitance of their transduction elements. As the sensing structure is miniaturized, the available capacitance decreases, so sensing performance can be degraded [13,14]. In addition, piezoresistive acoustic sensors employ a single-diaphragm structure, thereby avoiding issues associated with the air-gap structure of capacitive sensors such as ingress of moisture or dust, pull-in instability and stiction [15,16,17]. Furthermore, doped silicon itself can function as a piezoresistor, which reduces material and fabrication constraints compared with piezoelectric materials [18,19]. Despite these advantages, piezoresistive acoustic sensors have not been commercialized because of their insufficient sensitivity. Only piezoresistive pressure sensors intended for high-pressure applications have been commercialized.

To improve the existing limitation of piezoresistive acoustic sensors, there is an approach that replaces the conventional piezoresistor with a MOSFET with a piezoresistive channel (PrFET) [20]. A PrFET sensor is expected to improve its sensitivity, as a MOSFET has high input impedance and low output impedance so various signal conditioning methods are applicable [21,22,23]. Also, integration compatibility with CMOS circuits is expected to be improved, which facilitates functional density, low power operation, and miniaturization. The feasibility of PrFET devices for acoustic sensing has been demonstrated in previous research [24].

Table 1 summarizes recently reported studies on piezoresistive MEMS acoustic sensors, including their sensing structures, sensing areas, resonance frequencies, sensitivities, and acoustic overload point. Previous studies have explored various piezoresistive elements, including metal-induced piezoresistors, silicon nanowires, and graphene piezoresistors [25,26,27]. However, the PrFET acoustic sensor achieved the highest sensitivity among the previously reported piezoresistive acoustic sensors, demonstrating its potential to overcome the sensitivity limitations of conventional piezoresistive acoustic sensors. This previous study demonstrated the flat response over 18.07 Hz to 5.31 kHz. However, only one study on PrFET acoustic sensors has been reported to date, no studies have attempted to achieve enhanced sensitivity within a specific frequency range.

For the improvement or diversification of acoustic response characteristics, geometry and structural configuration have been actively exploited for various acoustic devices, including topological acoustic switches [28], resonant sensors [29] and acoustic sensors [30]. In particular, cantilever diaphragms have been applied to acoustic sensors for the purpose of sensitivity improvement within a specific range. A cantilever’s free end allows it to move with a large displacement, so its mechanical compliance can be high [31]. In piezoelectric sensors, the high stiffness of the piezoelectric layer can limit the mechanical response of the diaphragm when an edge-clamped structure is employed. Therefore, sector-shaped diaphragms formed by introducing slits in the center have been widely used, as they enhance the mechanical response without a substantial increase in the size of the diaphragm [32]. Additionally, in some applications that require monitoring a specific frequency, cantilever diaphragms have been investigated for various kinds of acoustic sensors such as photoacoustic [33,34,35], and piezoresistive type sensors [31,36,37,38].

This paper aims to investigate the effect of a cantilever diaphragm structure on the sensing characteristics of a PrFET acoustic sensor. For this purpose, the four-anchored circular diaphragm used in the previously reported PrFET acoustic sensor was modified into a sector-shaped cantilever structure. The cantilever diaphragm was designed based on its resonance frequency to achieve enhanced sensitivity within a specific frequency range below the resonance. Then, the frequency response of the proposed sensor was experimentally validated. The results demonstrate the potential for developing PrFET acoustic sensors with various response characteristics for diverse applications.

## 2. Design and Analysis

### 2.1. Signal Response of PrFET Acoustic Sensor

**Figure 1:**
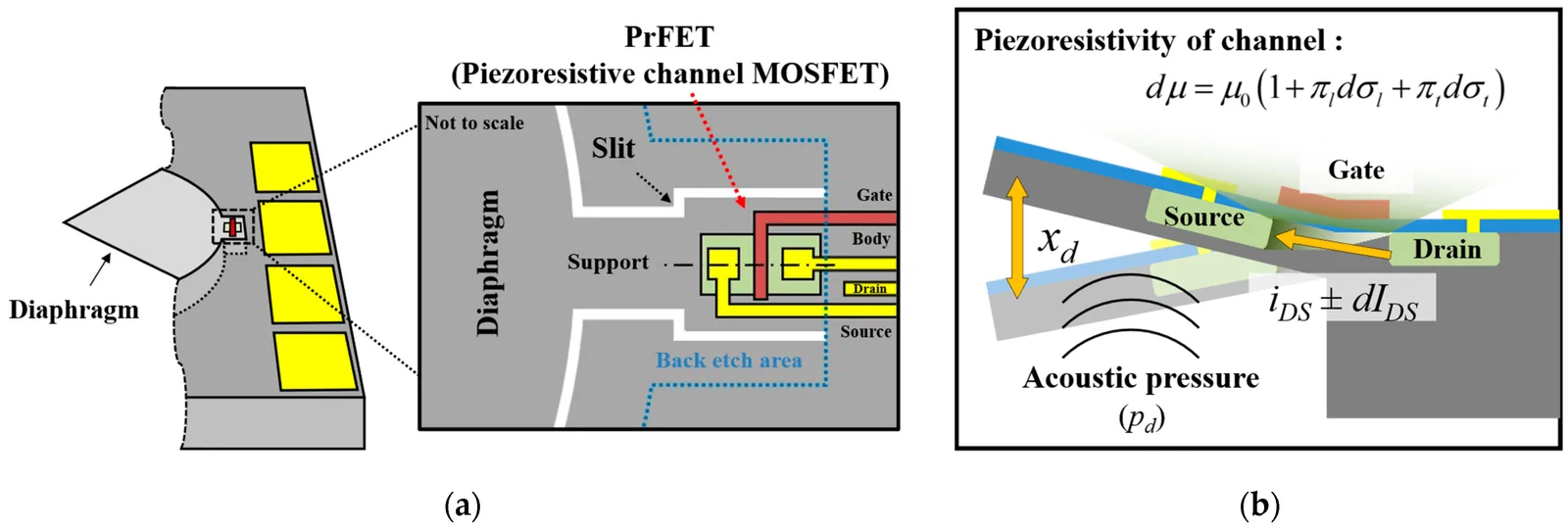
Schematic of the PrFET acoustic sensor: (**a**) structure and component, and (**b**) cross-section of the sensing region during operation.

The sensing principle of the PrFET acoustic sensor is based on the piezoresistive effect in the MOSFET channel. Figure 1a shows a schematic of the PrFET acoustic sensor. The sensor is composed of a diaphragm and a MOSFET embedded near the clamped boundary. Figure 1b describes the sensing mechanism. When acoustic pressure (*p_d_*) is incident on the diaphragm, stress is induced in the MOSFET channel. In the saturation region, the MOSFET channel current is determined by the channel properties, including carrier mobility, and the operating conditions, as follows [39].

(1) $$I_{\mathrm{DS}}=\frac{1}{2}\mu C_{\mathrm{ox}}\frac{W}{L}{\left(V_{\mathrm{GS}}-V_{T}\right)}^{2}$$

where *C_ox_* is the capacitance of the gate oxide per unit area, *μ* is the carrier mobility, *L* and *W* are the length and width of the channel, respectively, and *V_GS_* and *V_T_* are the gate and threshold voltages of the MOSFET.

In the case of a single-crystal silicon channel, the piezoresistive effect occurs independently under longitudinal and transverse stresses [40]. The carrier mobility *μ* under pressure excitation can be expressed as follows:

(2) $$\frac{\mathrm{d\mu}}{dp_{d}}=\mu_{0}\left(1+\pi_{l}\frac{d\sigma_{l}}{dp_{d}}+\pi_{t}\frac{d\sigma_{t}}{dp_{d}}\right)$$

where *μ*_0_ is the mobility under pre-stress, *σ_l_* and *σ_t_* are the longitudinal and transverse stresses, respectively, and *π_l_* and *π_t_* are the longitudinal and transverse piezoresistive coefficients. For the n-type channel, these values have been reported as *π_l_* = −3.55 × 10^−10^ Pa^−1^ and *π_t_* = −1.45 × 10^−10^ Pa^−1^ [41]. The bending stress in a diaphragm has been assumed to be plane stress, and shear stress is regarded as negligible [42].

The channel current change with respect to pressure (*dI_DS_*/*dp_d_*) is determined by the mobility changes with respect to pressure. When the constant term *μ*_0_*C_ox_*(*W*/*L*) is defined as the transconductance parameter *K_n_*, the current response is represented as follows:

(3) $$\frac{dI_{\mathrm{DS}}}{dp_{d}}=\frac{1}{2}K_{n}{\left(V_{\mathrm{GS}}-V_{T}\right)}^{2}\times \left(1+\pi_{l}\frac{d\sigma_{l}}{dp_{d}}+\pi_{t}\frac{d\sigma_{t}}{dp_{d}}\right)$$

Considering each parameter, *K_n_*, *V_T_*, *π_l_*, and *π_t_* are determined by the MOSFET characteristics, while *V_GS_* is determined by the MOSFET operating conditions. These parameters can be regarded as frequency-independent terms. Therefore, the frequency-response characteristic of the channel current signal is determined by the terms *dσ_l_*/*dp_d_* and *dσ_t_*/*dp_d_*, which refer to the stress responses to the acoustic input. These terms represent acousto-mechanical transduction and are determined by the mechanical characteristics of the diaphragm.

### 2.2. Design and Analysis of Cantilever Diaphragm

#### 2.2.1. Structural Design of the Diaphragm

**Figure 2:**
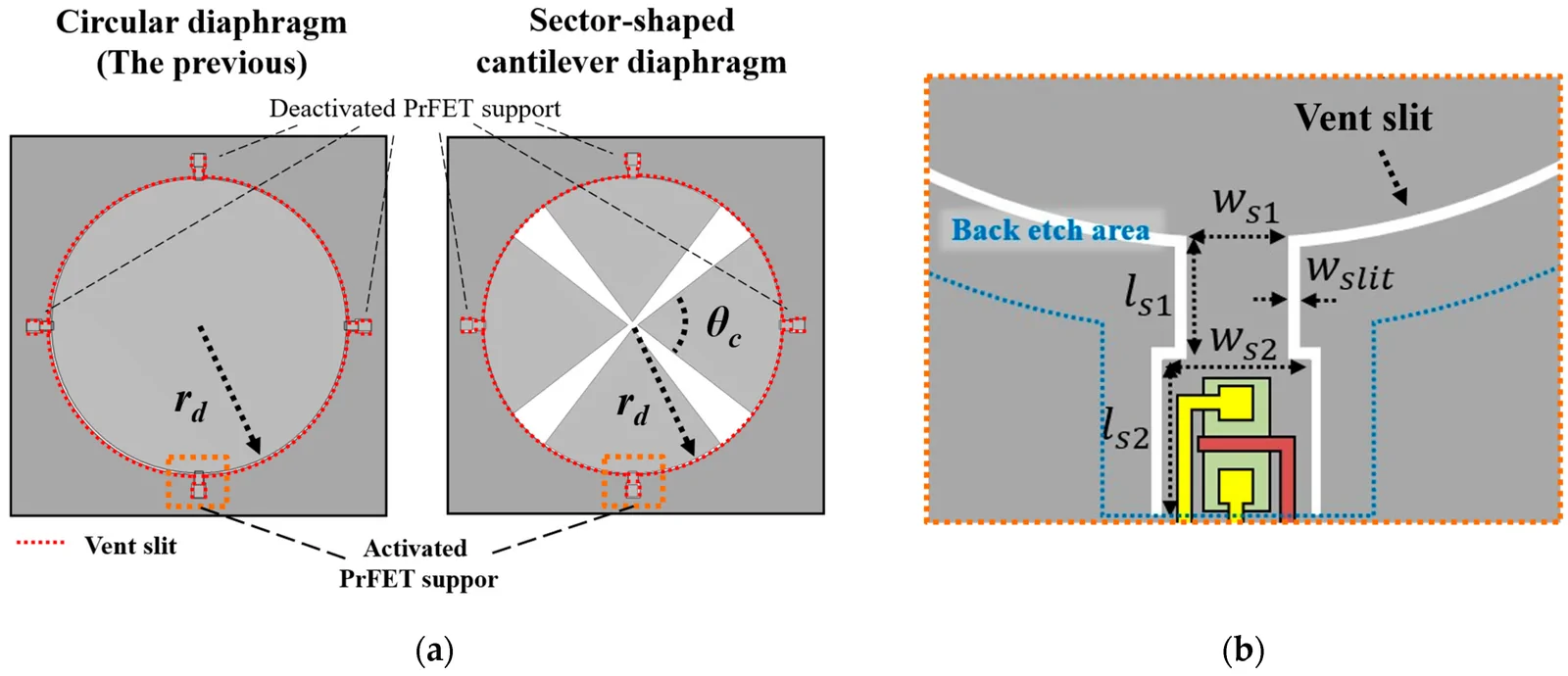
(**a**) Shapes of the circular and sector-shaped cantilever diaphragm and (**b**) magnified view and geometric parameters of the MOSFET-embedded support.

Figure 2 presents the circular diaphragm used in the previous PrFET acoustic sensor and the sector-shaped cantilever diaphragm proposed in this study. The previous circular diaphragm on the left side of Figure 2a is anchored by four MOSFET-embedded supports, and the edge is vented except at the supports [24]. The diaphragm is a bilayer composite of 2.2 μm of silicon and a 0.48 μm silicon dioxide layer. Of the four MOSFETs located at the supports, only one was activated for signal acquisition, while the remaining three were deactivated.

The right side of Figure 2a shows the sector-shaped cantilever diaphragm. The previous circular diaphragm was divided into four sector-shaped cantilevers, and each cantilever is anchored by a single support and has a free end at the center. The sector-shaped cantilever diaphragm’s thickness, radius, and MOSFET-embedded support geometry are identical to those of the circular diaphragm. Figure 2b shows a magnified view of the diaphragm support in which the PrFET is embedded. The geometric parameters shared by the circular and sector-shaped diaphragms are listed in Table 2.

**Figure 3:**
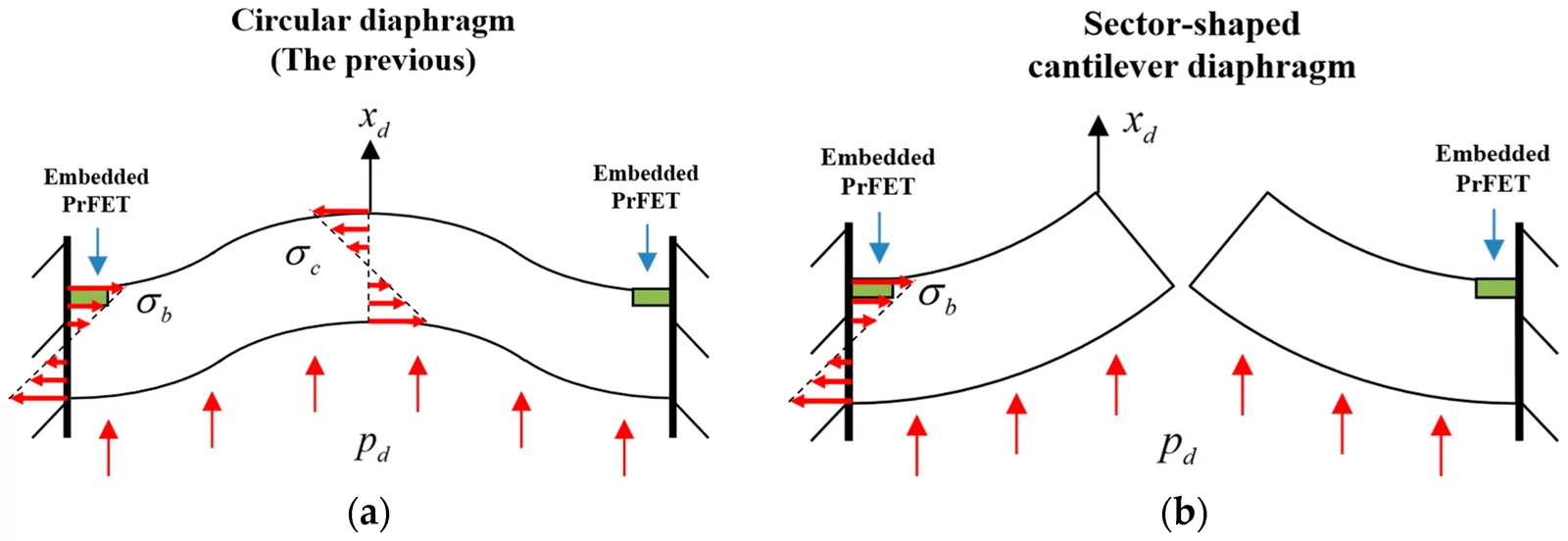
Schematic of cross-section view of deformation by pressure of (**a**) the circular diaphragm and (**b**) sector-shaped cantilever diaphragm.

Figure 3 shows schematic cross-sectional views of the diaphragms deformed by acoustic pressure. The PrFET is embedded at the clamped end of each structure, and the signal response is determined by the stress induced at this location. The circular diaphragm is clamped at each end. Therefore, bending stresses *σ_b_* and *σ_c_* are induced at the boundary ends and the center, respectively. In contrast, the newly designed sector-shaped cantilever diaphragm is clamped at the outer edge and has a free end toward the center. While the bending stress *σ_b_* is induced at the clamped boundary, the stress at the central free end is released. Due to this change in the boundary conditions, the sector-shaped cantilever diaphragm can induce greater stress under acoustic excitation of the PrFET. This can increase the current signal caused by the piezoresistive effect in the MOSFET channel.

For the circular diaphragm, the characterization of the mechanical response, including the stress induced by acoustic excitation, was conducted in the previous study [24]. The PrFET acoustic sensor incorporating this diaphragm was demonstrated to exhibit a flat response over the frequency range from 18.07 Hz to 5.31 kHz. In this study, the mechanical characteristics of the sector-shaped cantilever diaphragm, through which acoustic input induces stress in the PrFET, are investigated. Then, the frequency-response characteristics of the PrFET acoustic sensor incorporating this diaphragm are designed and experimentally validated.

#### 2.2.2. Characterization of the Sector-Shaped Cantilever

To determine the response characteristics of the PrFET acoustic sensor, the responses of the stresses to acoustic pressure, *dσ_l_*/*dp_d_* and *dσ_t_*/*dp_d_*, in the frequency domain must be characterized. They can be considered as a series of pressure-to-displacement and displacement-to-stress characteristics, as follows:

(4) $$\frac{d\sigma_{l}}{dp_{d}}=\frac{d\sigma_{l}}{dx_{d}}\times \frac{dx_{d}}{dp_{d}}$$

(5) $$\frac{d\sigma_{t}}{dp_{d}}=\frac{d\sigma_{t}}{dx_{d}}\times \frac{dx_{d}}{dp_{d}}$$

Here, *dσ_l_*/*dx_d_* and *dσ_t_*/*dx_d_* represent the stress induced per unit displacement of the diaphragm. Assuming small, linear-elastic deformation of the diaphragm, these terms can be treated as constant values, as governed by Hooke’s law. The remaining term, *dx_d_*/*dp_d_*, represents the displacement response to the acoustic input. Both the parameters associated with the electrical characteristics of the MOSFET and the displacement-to-stress characteristics can be regarded as frequency-independent. Consequently, the frequency-response characteristics of the PrFET sensor are primarily determined by the design of the diaphragm’s displacement response to the acoustic input.

To characterize the frequency-dependent displacement response *dx_d_*/*dp_d_*, the diaphragm was modeled as an equivalent single-degree-of-freedom (1-DOF) mechanical system, composed of a lumped spring, mass and damper. Assuming that the acoustic pressure incident on the diaphragm, *p_d_*, and the resulting displacement *x_d_* are harmonic signals with angular frequency *ω*, the displacement response to the acoustic pressure can be expressed as follows:

(6) $$\frac{dx_{d}}{dp_{d}}=\frac{1}{\mathrm{j\omega}A_{d}}\times \frac{1}{\mathrm{j\omega}\frac{m_{d}}{{A_{d}}^{2}}+\frac{k_{d}}{\mathrm{j\omega}{A_{d}}^{2}}+\frac{R_{m,d}}{{A_{d}}^{2}}}$$

Here, *A_d_* is the diaphragm’s sensing area exposed to the acoustic pressure, *m_d_* is the lumped mass, *k_d_* is the stiffness of the lumped spring and *R_m_*_,*d*_ is the damping coefficient of lumped damper of the diaphragm. From this expression, an equivalent circuit can be constructed by representing the incident acoustic pressure *p_d_* as an electrical potential and the diaphragm volume velocity *A_d_* (*dx_d_*/*dt*) as an electrical current. In this equivalent circuit, *m_d_*/*A_d_*^2^, *A_d_*^2^/*k_d_* and *R_m_*_,*d*_/*A_d_*^2^ can be represented as an equivalent inductance, capacitance and resistance, respectively, which are connected in series [8].

**Figure 4:**
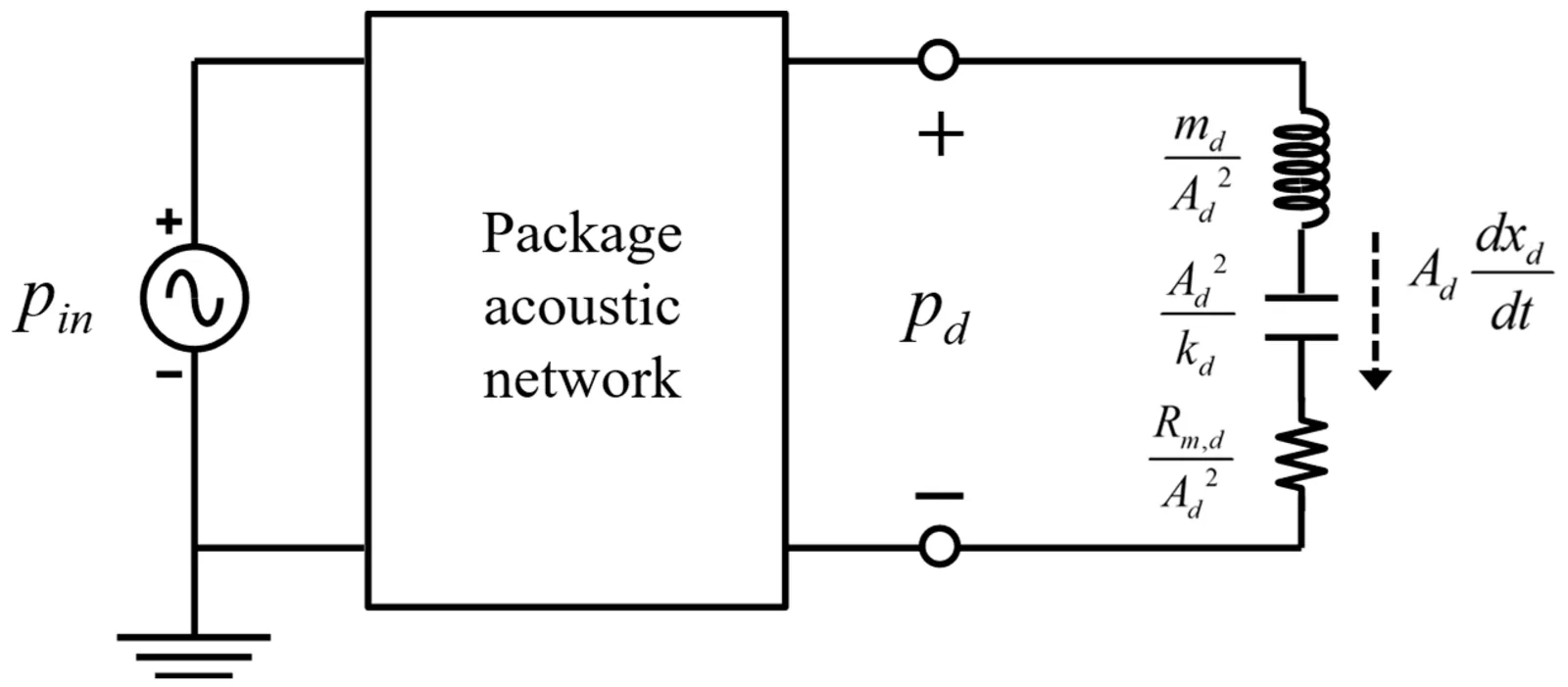
Equivalent acoustic circuit network of the PrFET acoustic sensor with the diaphragm contained in a package.

Acoustic sensor diaphragms are typically integrated into a package, so the incident pressure on the diaphragm is affected by the acoustic characteristics of the package. Figure 4 shows an equivalent-circuit model for the transmission of the sensor’s input acoustic pressure, *p_in_*, to the diaphragm through the acoustic structure of the package. In this representation, the acoustic effects of the package are represented by a package acoustic network located between the sensor input pressure, *p_in_*, and the diaphragm incident pressure, *p_d_*.

The mechanical characteristics of the diaphragm are represented by the parameters *A_d_*, *m_d_*, *k_d_* and *R_m_*_,*d*_. After these parameters are determined from the designed diaphragm, they can be combined with the equivalent circuit incorporating the characterized acoustic network of the package. This enables the displacement response of the diaphragm to the actual input acoustic pressure, *p_in_*, to be modeled.

**Figure 5:**
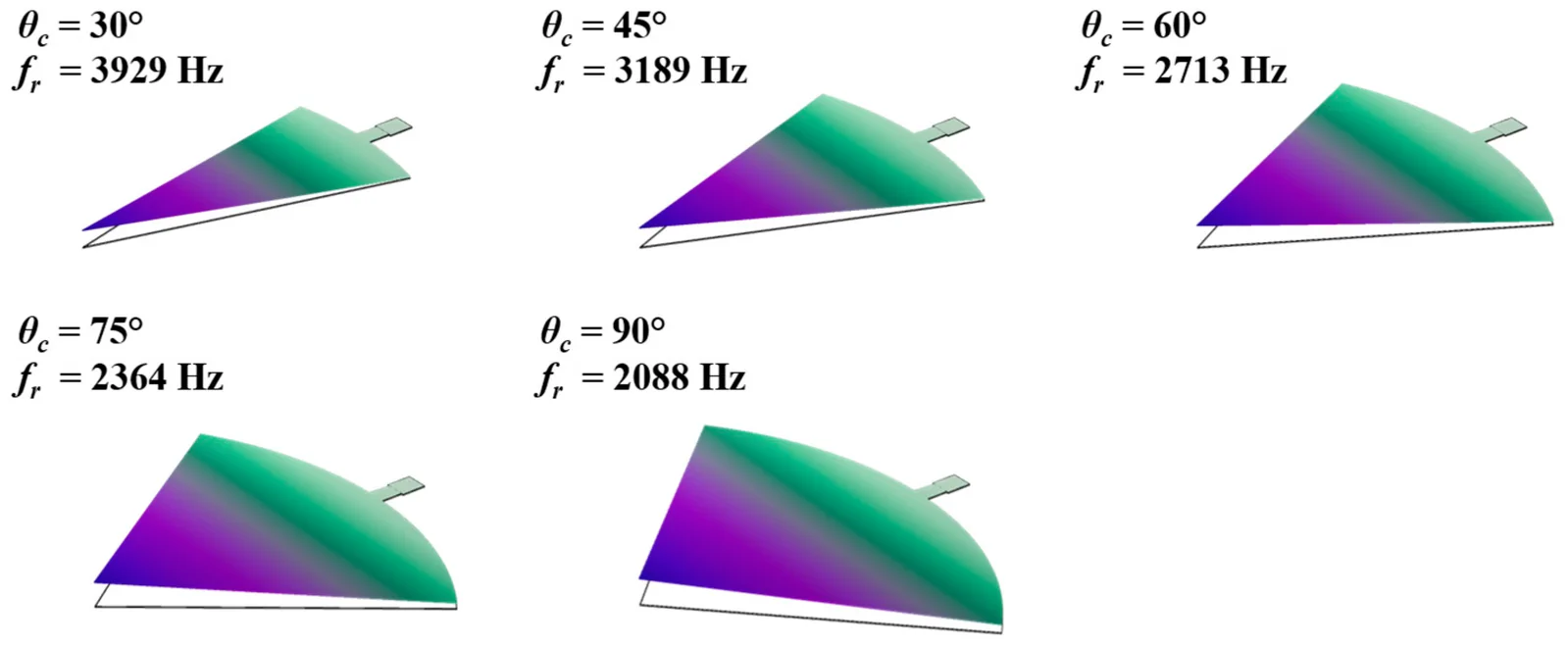
Mode shapes and frequencies of first resonance of the cantilever diaphragms with central angles of 30°, 45°, 60°, 75°, and 90°.

To analyze the available range of resonance frequencies with this sector-shaped cantilever structure, its central angle (*θ_c_*) was varied. The mechanical response characteristics were compared for different central angles: *θ_c_* = 30°, 45°, 60°, 75° and 90°. The first resonance mode for each central angle was analyzed using an eigenfrequency study in COMSOL Multiphysics 5.6. Figure 5 presents the first-mode shape and corresponding resonance frequency for each central angle. The analysis confirmed that the proposed cantilever structure can exhibit resonance frequencies ranging from 2088 to 3929 Hz by varying the central angle.

- Displacement-to-stresses characterization (*dσ_l_*/*dx_d_* & *dσ_t_*/*dx_d_*)

**Figure 6:**
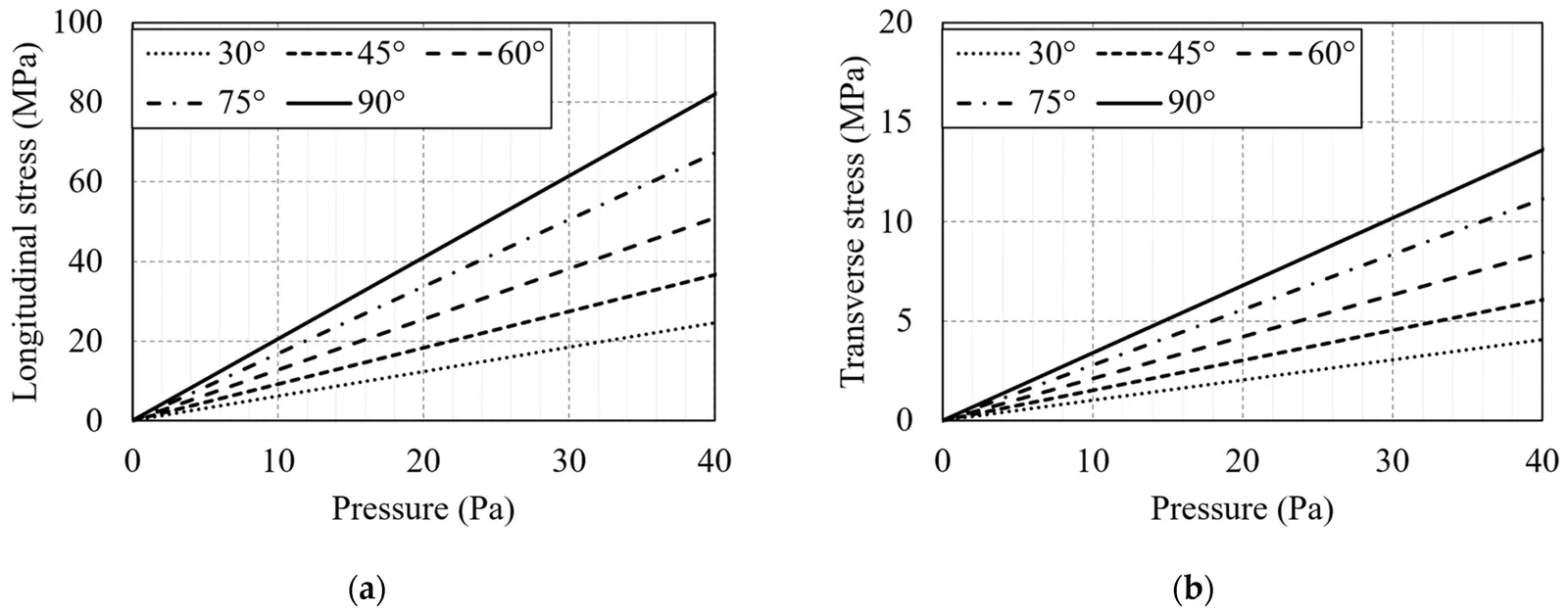
Calculated (**a**) longitudinal and (**b**) transverse stresses at the center of the PrFET channel depending on the static pressure acting on the diaphragm.

**Figure 7:**
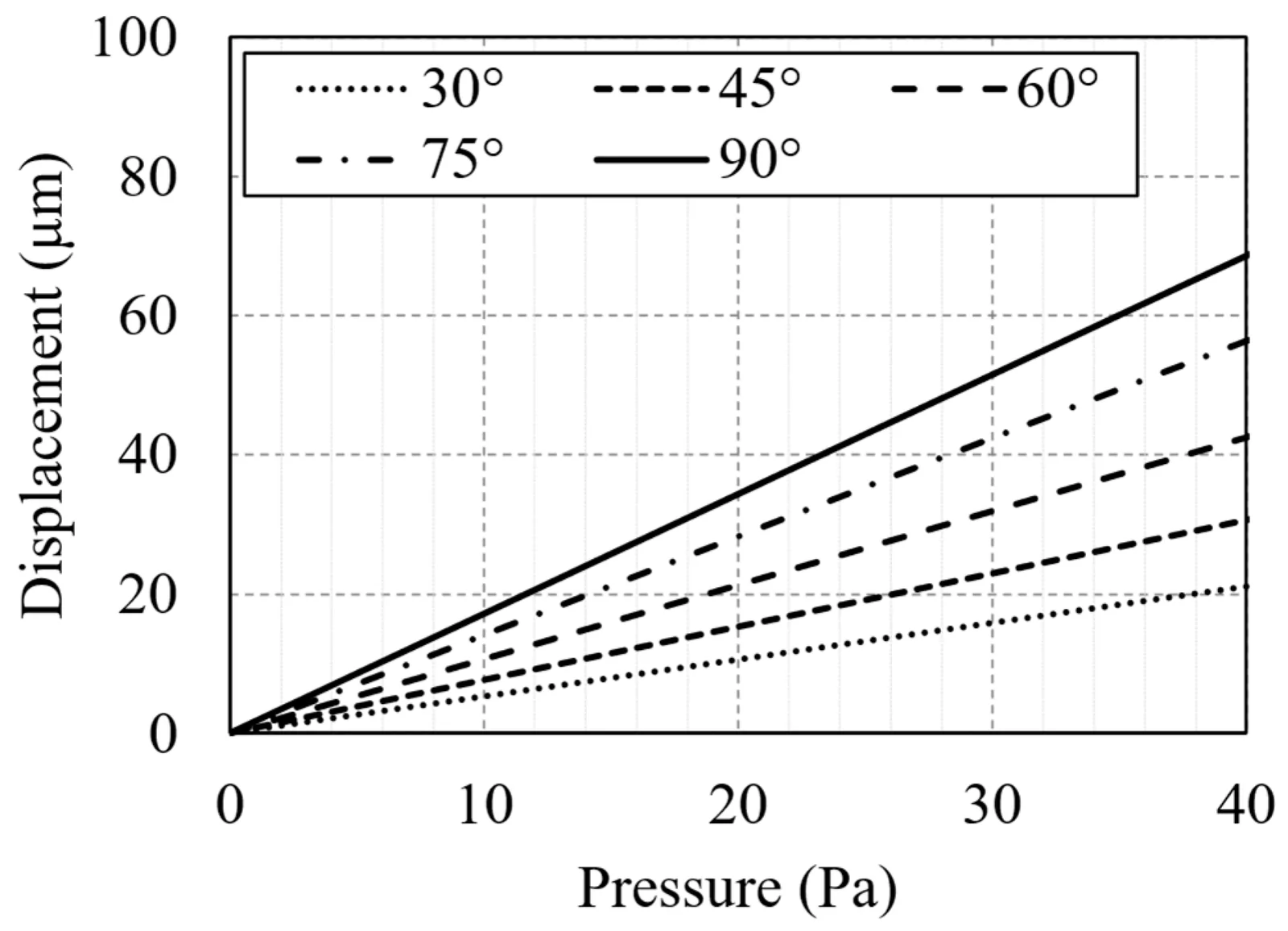
Calculated displacement at the center of cantilever depending on the static pressure acting on the diaphragm.

The displacement-to-stress characteristics, *dσ_l_*/*dx_d_* and *dσ_t_*/*dx_d_* were evaluated using simulations of the longitudinal and transverse stresses and the displacement at the diaphragm center under the applied pressure. The simulations were conducted using COMSOL Multiphysics 5.6 under static pressure loading, with geometric nonlinearity included in the solver. Figure 6 presents the calculated longitudinal and transverse stresses in the PrFET channel as functions of the applied pressure. Figure 7 presents the calculated center displacement as a function of the applied pressure.

The analysis was conducted over a pressure range from 0 to 40 Pa. Although geometric nonlinearity was considered in the analysis, both the stress and displacement were found to increase approximately linearly within this range. This indicates that the diaphragm exhibits an approximately linear mechanical response within the considered pressure range, allowing the relationship between displacement and stress to be characterized by constant proportionality coefficients. In addition, larger central angles resulted in greater displacement and stress. Comparison among the different central angles showed that the stress-to-displacement ratios remained nearly identical for all angles. The displacement-to-stress ratios were determined to be *dσ_l_*/*dx_d_* = 1.19 × 10^12^ Pa/m and *dσ_t_*/*dx_d_* = 0.19 × 10^12^ Pa/m.

- Pressure-to-displacement response characterization (*dx_d_*/*dp_d_*)

Each of the diaphragm’s parameters, *A_d_*, *k_d_* and *m_d_* is characterized as follows. First, the sensing area, *A_d_*, can be directly determined from the geometry of each cantilever. Next, the stiffness, *k_d_*, was defined as the force per unit displacement at the center of the diaphragm in this study. This can therefore be obtained from the pressure–displacement relationship already presented in Figure 7 by converting the applied pressure into the corresponding force.

For the mass, *m_d_*, directly using the physical mass of the cantilever does not accurately represent its dynamic behavior. Instead, when the stiffness and natural frequency (*f_n_*) of a single-degree-of-freedom system are known, the effective mass can be derived from the following relationship:

(7) $$m_{d}=\frac{k_{d}}{{\left(2\pi f_{n}\right)}^{2}}$$

The natural frequencies correspond to the first mode frequencies presented in Figure 5, and *k_d_* can be derived using the method described above.

The damping coefficient, *R_m_*_,*d*_, is related to energy dissipation, and the dissipation of vibrating structures occurs due to thermoelastic damping. For beam structures vibrating in flexural modes, the quality factor associated with thermoelastic damping, *Q_TED_*, is described by Zener’s model as follows [43]:

(8) $$\frac{1}{Q_{\mathrm{TED}}}=\frac{E\alpha^{2}T_{0}}{C_{P}}\frac{\omega \tau_{Z}}{1+{\left(\omega \tau_{Z}\right)}^{2}}$$

where *τ*_Z_ is the thermal relaxation time and is expressed as

(9) $$\tau_{Z}=\frac{\rho_{b}C_{P}{t_{b}}^{2}}{\pi^{2}\kappa_{\mathrm{th}}}$$

In (8) and (9), *E* is Young’s modulus, α is the linear thermal expansion coefficient, *T*_0_ is the equilibrium temperature, *C_P_* is the specific heat capacity, *χ* is the thermal diffusivity and *t_b_* is the thickness of the beam and *ω* is the angular frequency.

The effective quality factor of the cantilever, *Q_eff_*, is considered as follows:

(10) $$\frac{1}{Q_{\mathrm{eff}}}=\frac{1}{t_{\mathrm{Si}}+t_{\mathrm{Si}O_{2}}}\times {\left(\frac{t_{\mathrm{Si}}}{Q_{\mathrm{Si}}}+\frac{t_{\mathrm{Si}O_{2}}}{Q_{\mathrm{Si}O_{2}}}\right)}^{-1}$$

where *t_Si_* and *t_SiO_*_2_ are the thicknesses, and *Q_Si_* and *Q_SiO_*_2_ are the thermoelastic damping quality factors of the silicon layer and the silicon dioxide layer, respectively. Next, the damping coefficients of each angle are estimated as follows:

(11) $$R_{m,d}=\frac{\sqrt{k_{d}m_{d}}}{Q_{\mathrm{eff}}}$$

The designed sector-shaped cantilever is a bilayer structure composed of silicon and silicon dioxide. Each material’s reported properties and dimensions are listed in Table 3.

Table 4 lists the derived parameters associated with the pressure-to-displacement response.

**Figure 8:**
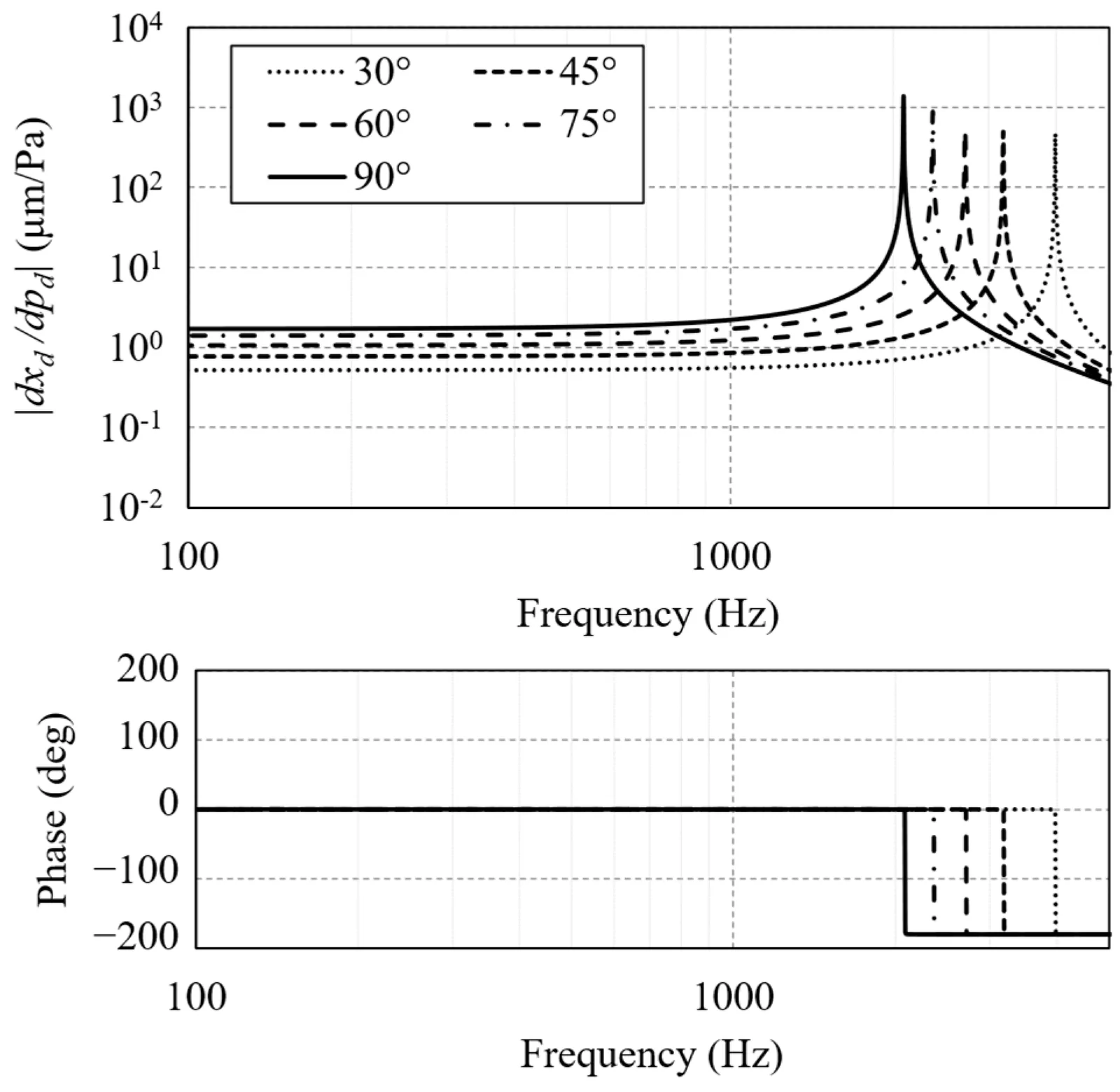
Magnitude and phase frequency response of displacement by diaphragm incident pressure.

Based on the derived parameters, the frequency response from incident pressure to displacement is presented in Figure 8, as expressed in Equation (6). Analysis of the cantilever diaphragms showed that each diaphragm exhibited a flat displacement response below its resonance frequency. As the central angle increases, the resonance frequency decreases, while the response level in the flat frequency range below resonance increases. Therefore, the central angle of the sector-shaped cantilever can serve as a design parameter for determining both the response level and the bandwidth of the flat response region. Among the investigated designs, the diaphragm with a central angle of 90° exhibited the highest displacement response within the flat-response region. In this study, the central angle of 90° was selected for experimental validation of the signal response.

#### 2.2.3. Diaphragm Response by Sensor Input Pressure

**Figure 9:**
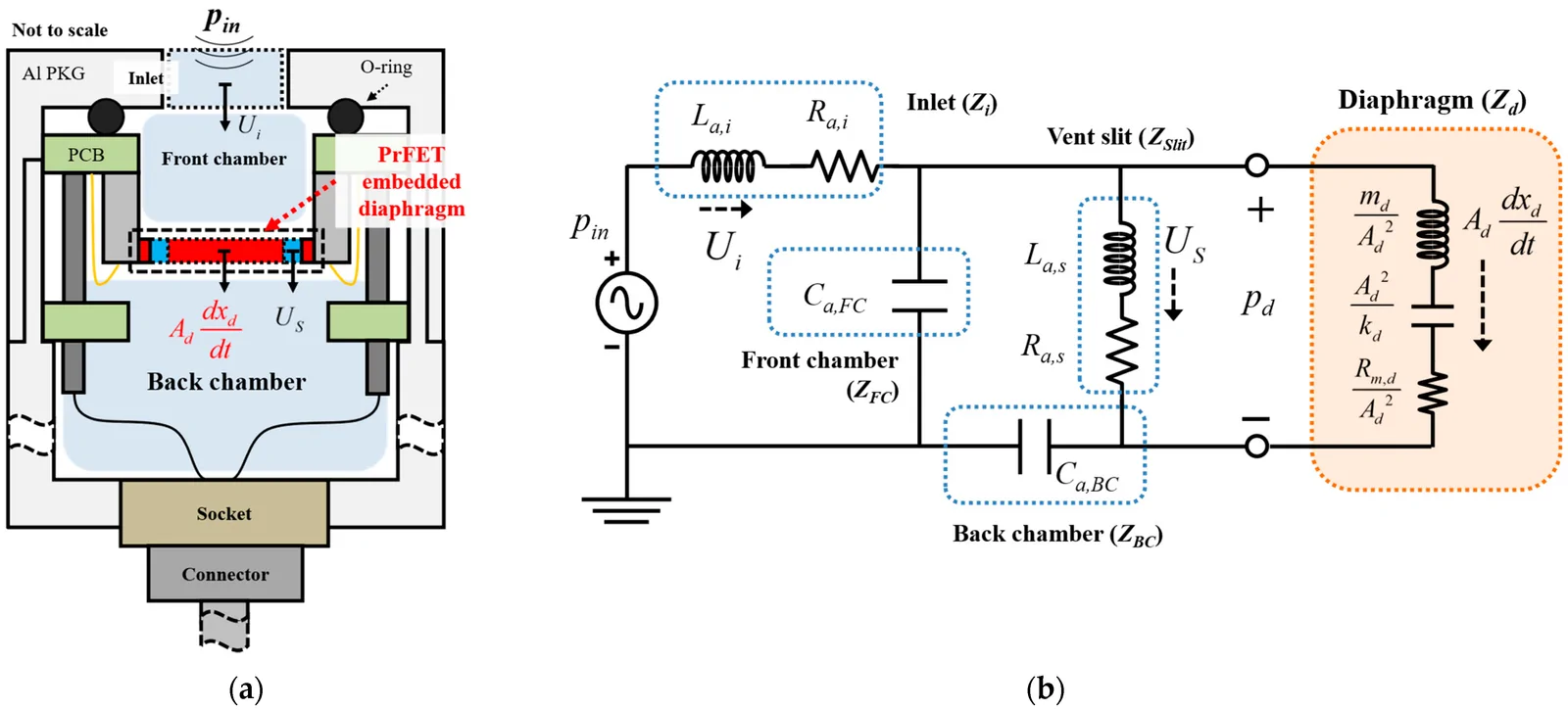
(**a**) Schematic of PrFET acoustic sensor package. (**b**) Equivalent acoustic circuit incorporated with the package acoustic network.

The displacement response to the sensor input pressure, *p_in_*, applied to the sensor system is represented by *dx_d_*/*dp_in_*. Determination of this term should consider the acoustic network of the package through which *p_in_* is transmitted. The sensor package used in this study is the same as that employed in the previously reported PrFET acoustic sensor [24]. A schematic of the package structure and the equivalent-circuit model incorporating the package acoustic network are shown in Figure 9.

Figure 9a presents the schematic of the package case that contains an inlet hole, a front chamber and back chamber. The input acoustic pressure enters through the inlet hole, is transmitted to the diaphragm through the front chamber, and then passes through the vent slit into the back chamber. In Figure 9b, the acoustic pressure is represented as an alternating source, and the volume velocities of airflow within the inlet hole and vent slit are denoted as *U_i_* and *U_s_*, respectively. The acoustic impedances of each structure are represented as lumped parameters using electrical-element analogies. The inlet hole and vent slit are modeled as acoustic inertances, *L_a_*_,*i*_ and *L_a_*_,*s*_ and resistances *R_a_*_,*i*_ and *R_a_*_,*s*_, respectively. The front chamber and back chamber have been modeled as acoustic compliances, *C_a_*_,*FC*_ and *C_a_*_,*BC*_, respectively. For convenience of representation, the acoustic impedances of the individual structures are denoted by the symbols listed in Table 5.

From the equivalent circuit, three following equations related to the volume velocities can be obtained as follows:

(12) $$p_{\mathrm{in}}=\left(Z_{a,i}+Z_{\mathrm{FC}}\right)U_{i}-Z_{\mathrm{FC}}\left(A_{d}\frac{dx_{d}}{\mathrm{dt}}\right)-Z_{\mathrm{FC}}U_{s}$$

(13) $$0=Z_{\mathrm{FC}}U_{i}-\left(Z_{d}+Z_{\mathrm{FC}}+Z_{\mathrm{BC}}\right)\left(A_{d}\frac{dx_{d}}{\mathrm{dt}}\right)-\left(Z_{\mathrm{FC}}+Z_{\mathrm{BC}}\right)U_{s}$$

(14) $$0=Z_{\mathrm{FC}}U_{i}-\left(Z_{\mathrm{FC}}+Z_{\mathrm{BC}}\right)\left(A_{d}\frac{dx_{d}}{\mathrm{dt}}\right)-\left(\;Z_{a,s}+Z_{\mathrm{FC}}+Z_{\mathrm{BC}}\right)U_{s}$$

By solving (12)~(14), the displacement response by acoustic pressure input can be expressed as follows:

(15) $$\frac{dx_{d}}{dp_{\mathrm{in}}}=\frac{1}{\mathrm{j\omega}A_{d}}\times \frac{{Z_{d}}^{1}{Z_{\mathrm{in}}}^{-1}{Z_{\mathrm{BC}}}^{-1}}{\left(\begin{array}{c} \left({Z_{s}}^{-1}+{Z_{d}}^{-1}+{Z_{\mathrm{in}}}^{-1}+{Z_{\mathrm{FC}}}^{-1}\right)\\ \times \left({Z_{s}}^{-1}+{Z_{d}}^{-1}+{Z_{\mathrm{BC}}}^{-1}\right)-{\left({Z_{s}}^{-1}+{Z_{d}}^{-1}\right)}^{2} \end{array}\right)}$$

The value of each acoustic impedance component is reported in the previous paper except for the vent slit. The geometry of the vent slit is changed according to the diaphragm design. Therefore, the vent slit’s acoustic inertance, *L_a_*_,*s*_, and acoustic resistance, *R_a_*_,*s*_, in the cantilever diaphragm are estimated by following expressions, respectively [8]:

(16) $$L_{a,i}=\frac{6\rho_{0}t_{\mathrm{slit}}}{5l_{\mathrm{slit}}w_{\mathrm{slit}}}\left[{\mathrm{kg}/m}^{4}\right]$$

(17) $$R_{a,i}=\frac{12\mu t_{\mathrm{slit}}}{{w_{\mathrm{slit}}}^{3}l_{\mathrm{slit}}}\left[N\times {s/m}^{5}\right]$$

where *ρ*_0_ is air density and its value is 1.225 kg/m^3^. *μ* is the dynamic viscosity of air, with a value of 1.86 × 10^−5^ N × s/m^2^. *t_slit_*, *w_slit_*, *l_slit_* and are thickness, length and width of the vent slit. The respective values are *t_slit_* = 2.68 μm, *w_slit_* = 4 μm, and *l_slit_* = 7198 μm. The values of each acoustic impedance component are listed in Table 6.

**Figure 10:**
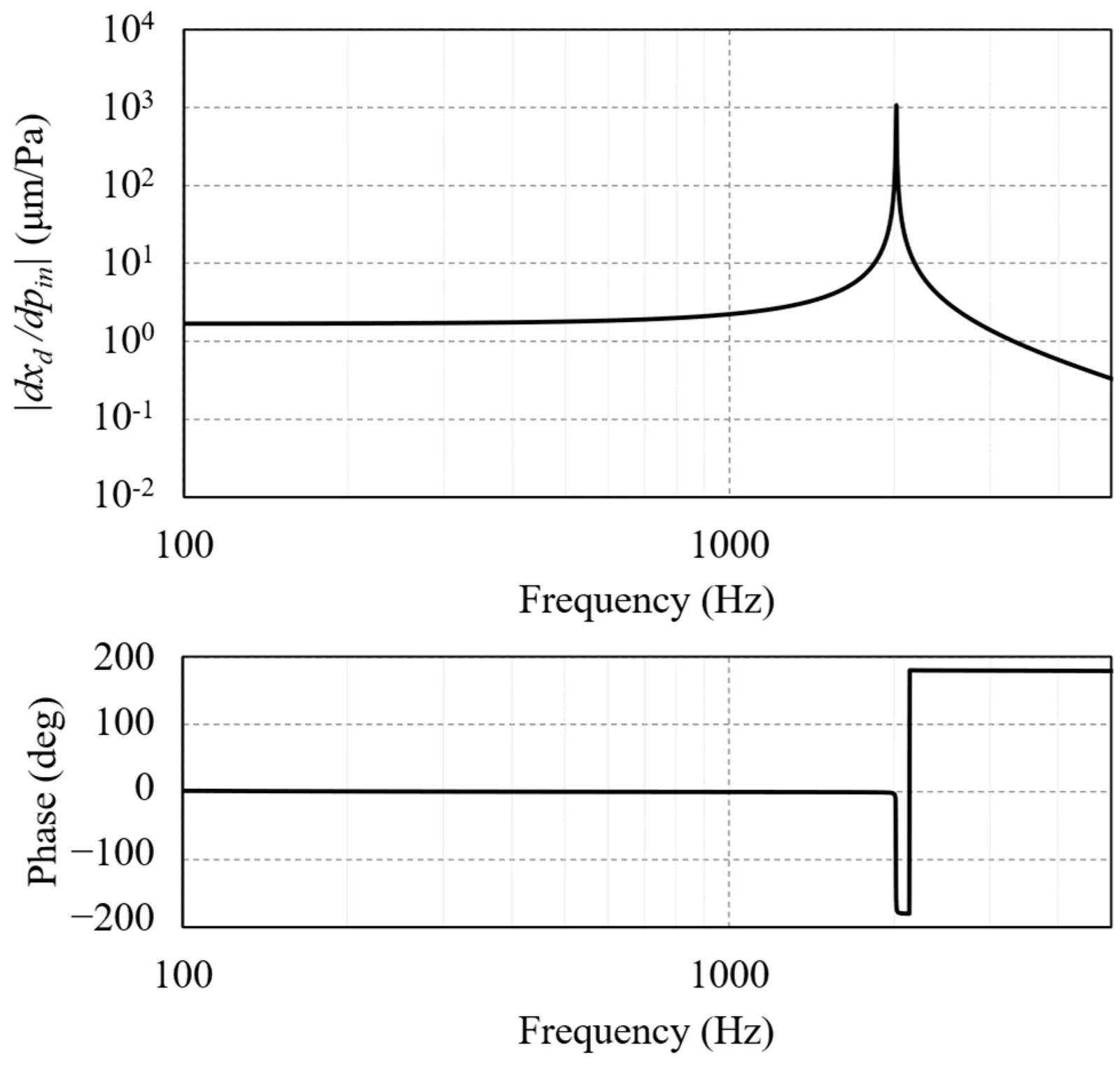
Magnitude and phase frequency response of displacement by sensor input pressure.

The calculated response is presented in Figure 10. A resonance is observed at 2.02 kHz, which is slightly lower than the natural frequency of the diaphragm (2.09 kHz). Since the frequency-response characteristics of the PrFET acoustic sensor are governed by the response obtained here. However, in the fabricated diaphragm, this resonance may shift due to effects such as residual stress. This effect is discussed later.

#### 2.2.4. Residual Stress Consideration

The diaphragm’s characteristics were analyzed without consideration of residual stress. However, the circular diaphragm fabricated in the previous PrFET acoustic sensor exhibited initial deformation due to residual stress induced during the deposition process [24]. Because initial stress can change the stiffness of the diaphragm, its effect must be considered [50]. Therefore, an FEM simulation was performed for the 90° cantilever design to evaluate the effect of initial stress.

**Figure 11:**
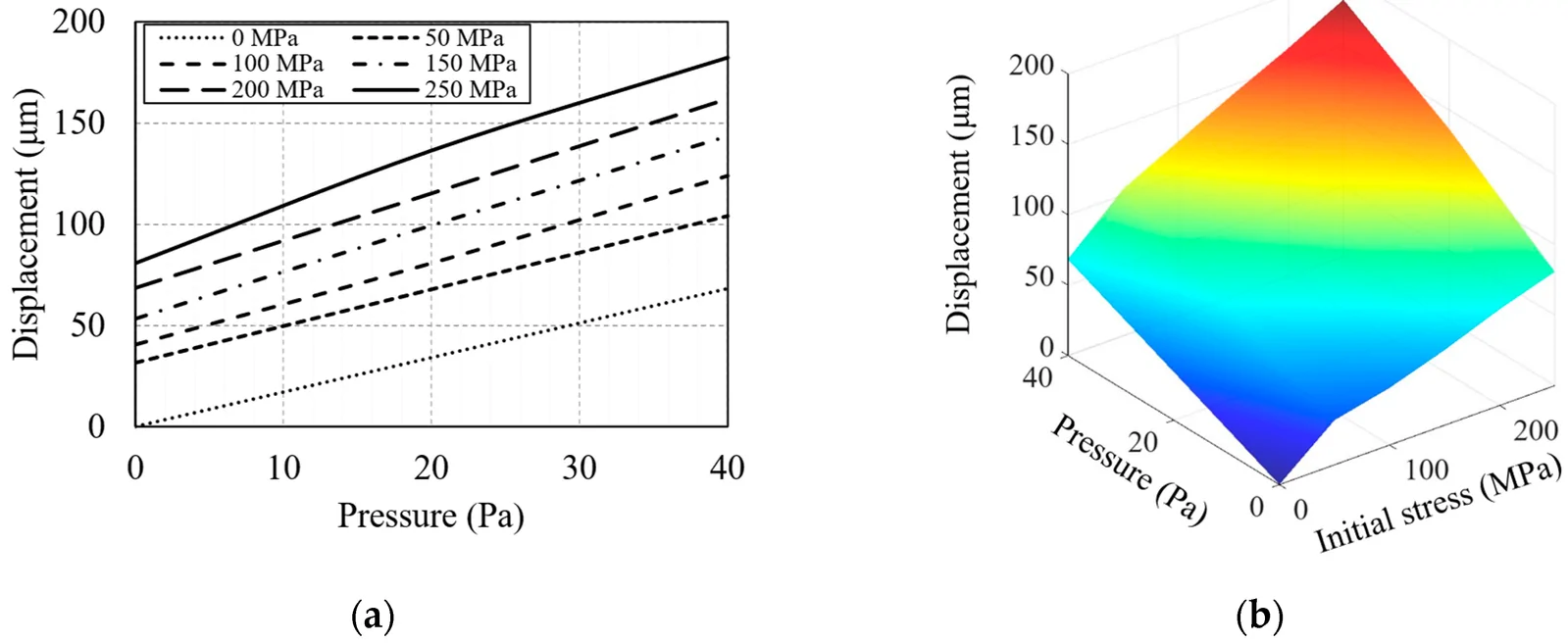
(**a**) A multiline plot and (**b**) 3D surface plot of the diaphragm’s initial stress, pressure and cantilever tip displacement.

Figure 11 presents the applied initial stress, pressure, and resulting displacement are presented in a multiline plot in Figure 11a and a 3D surface plot in Figure 11b. Using the FEM model used in Figure 6 and Figure 7, initial stress was applied to the silicon dioxide layer, and the tip displacement generated under the applied pressure was simulated. The simulation results show that the pressure-induced displacement varies depending on the initial stress. This suggests that the stiffness of the diaphragm can vary with the initial stress. Therefore, after fabrication, the residual stress was evaluated through post-fabrication characterization of the diaphragm, and the corresponding stiffness was subsequently recalculated.

## 3. Fabrication Process and Post-Fabrication Characterization

This section describes the fabrication processes and resulting characterization of the PrFET acoustic sensor with the designed cantilever diaphragm. The fabrication process consists of a CMOS process for MOSFET formation and a MEMS process for diaphragm formation. In this study, the MOSFET characteristics were intended to remain consistent with those of the previous study employing the circular diaphragm. Therefore, the CMOS fabrication process followed that reported in the previous study, and the characteristics of the fabricated MOSFET were subsequently analyzed [24]. In addition, the residual stress in the diaphragm fabricated through the MEMS process was evaluated to determine the diaphragm’s parameters.

### 3.1. MOSFET Characterization

**Figure 12:**
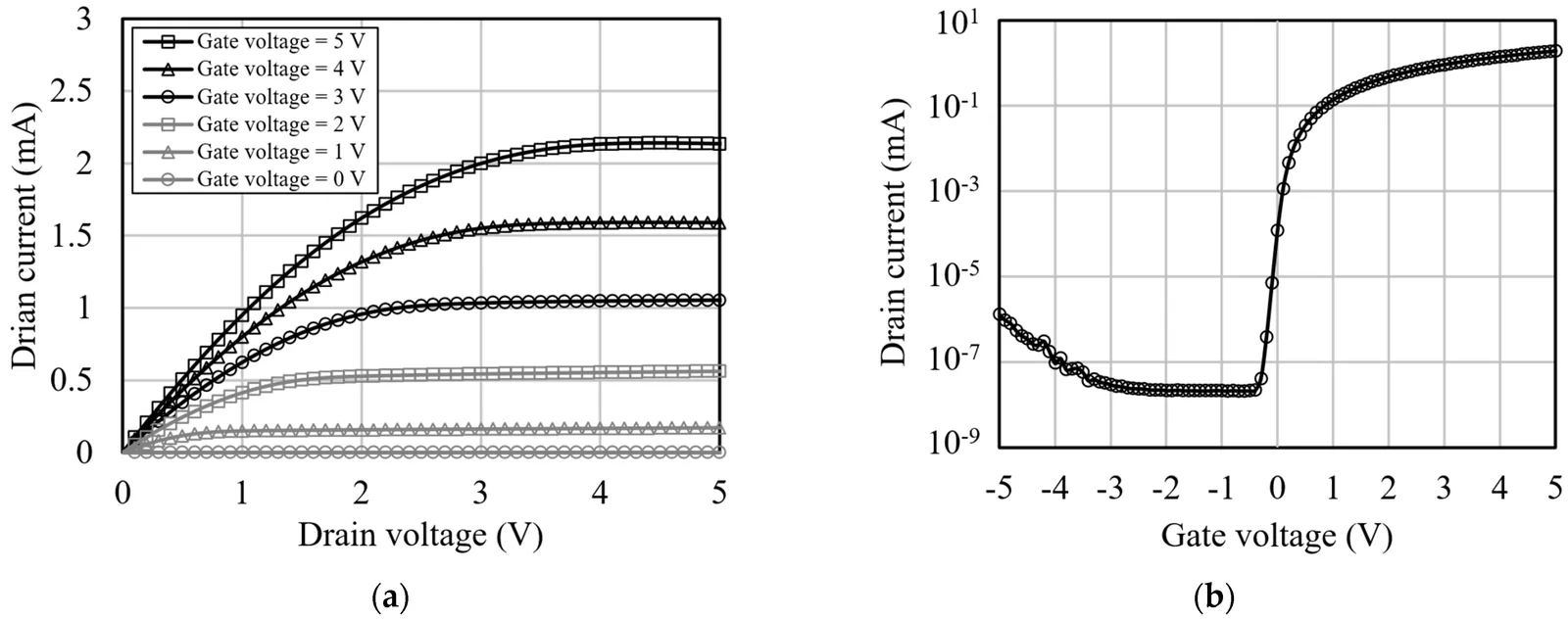
(**a**) *I_DS_*-*V_DS_* and (**b**) *I_DS_*-*V_GS_* (at *V_DS_* = 5 V) curves of the MOSFET.

Figure 12 shows the I-V curves of the PrFET fabricated by the CMOS process. These were obtained using a semiconductor analyzer (4200A-SCS; Keithley, Cleveland, OH, USA) that measured the source-drain current (*I_DS_*) during a voltage sweep at the wafer level. The MOSFET operated in a typical enhancement mode. The threshold voltage (*V_t_* = 1.2 V) was calculated by extrapolating the voltage at which the transconductance was maximum when the body voltage was zero and the source-drain voltage was 5 V. The transconductance parameters used for the calculations are listed in Table 7. These characteristics did not significantly differ from those used in the previous device with the circular diaphragm [24]. Therefore, the effect of the cantilever diaphragm on the sensor response could be analyzed while maintaining unchanged MOSFET characteristics.

### 3.2. MEMS Process and Residual Stress Characterization

**Figure 13:**
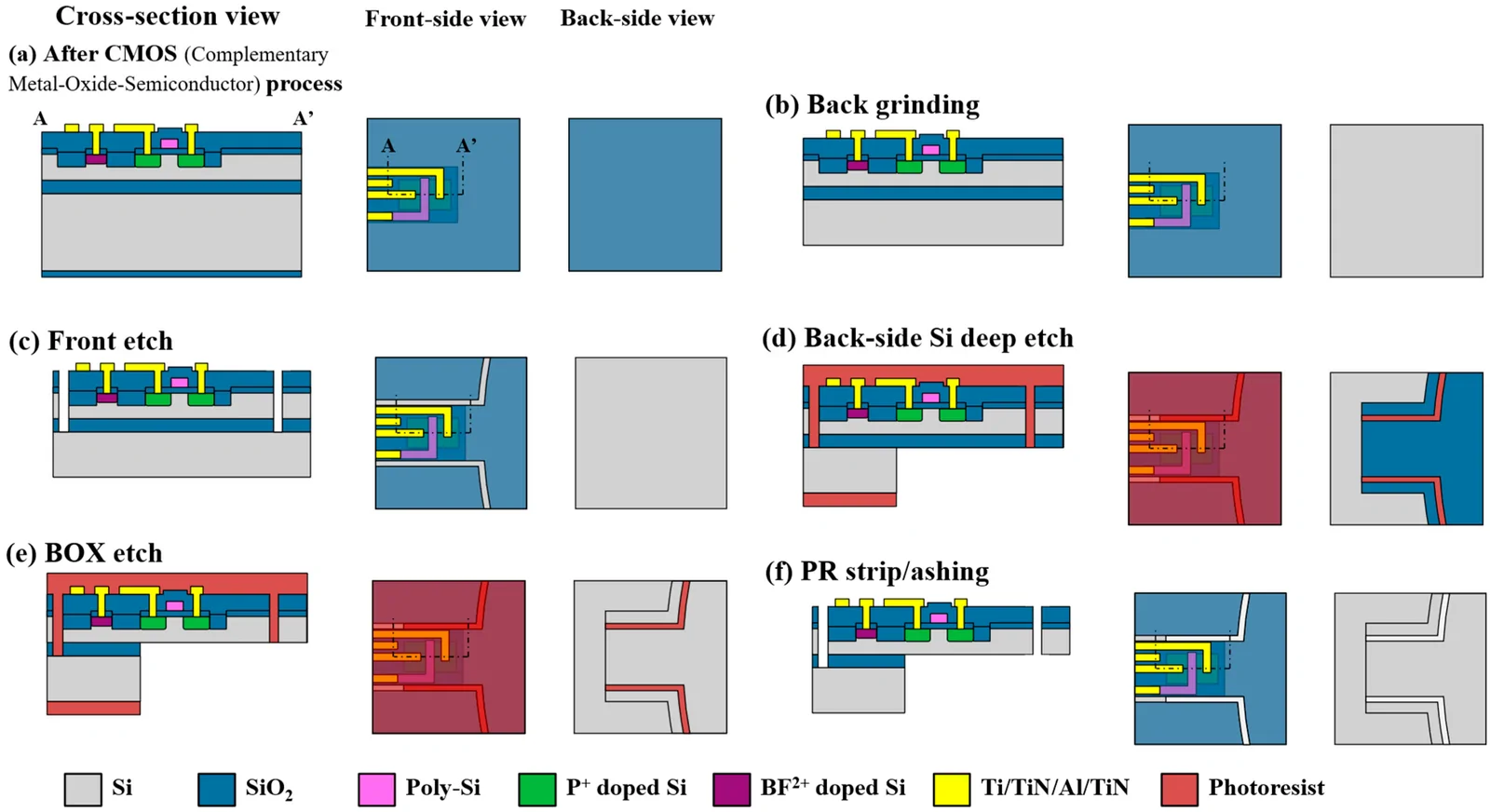
The MEMS process to fabricate the PrFET acoustic sensor with the cantilever diaphragm. (**a**) After the CMOS process. (**b**) Grinding of the handle layer on the back side. (**c**) Dry etching of the slit. (**d**) Front PR passivation and back-side DRIE. (**e**) BOX layer wet-etching using BOE. (**f**) PR removal with acetone, and ashing.

Figure 13 shows the MEMS process used to fabricate the cantilever diaphragm. Initially, the handle layer was ground on the back side of the wafer; the remaining layer was 407 μm in thickness (Figure 13b). The interlayer dielectric (ILD) (4500 Å), the gate oxide layer (300 Å), the silicon layer (2.2 μm), and the BOX layer (0.5 μm) were then etched according to the slit pattern for the sensing cantilever (Figure 13c). The cantilever was next created via deep reactive ion etching (DRIE) (Figure 13d). A 10 μm thick photoresist (PR) layer was then applied for front-side passivation, and the BOX layer was etched away using a wet buffered oxide etchant (BOE) (Figure 13e). The photoresist was next removed with acetone, followed by ashing, and individual acoustic sensor chips were obtained via blade-dicing.

**Figure 14:**
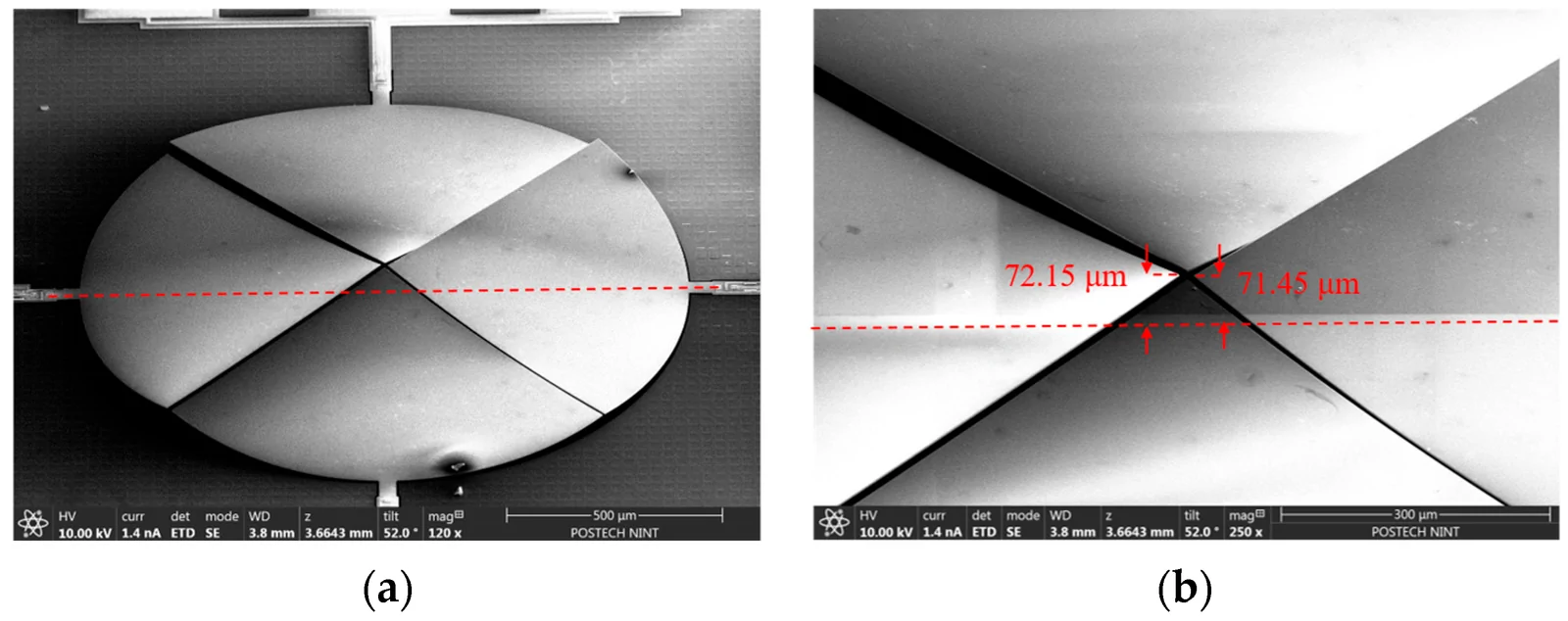
SEM image of cantilever diaphragm and measured displacement of cantilevers’ tip. The red dashed line indicates the reference level used to measure the vertical displacement of the cantilever tips.

Figure 14 shows SEM images of the fabricated diaphragm, which exhibited curvature due to residual stress in the deposited layer. As described in Section 2.2.4, the diaphragm characteristics can be changed by residual stress. The height of the cantilever’s tip increased, and the measured height averaged 71.8 μm.

**Figure 15:**
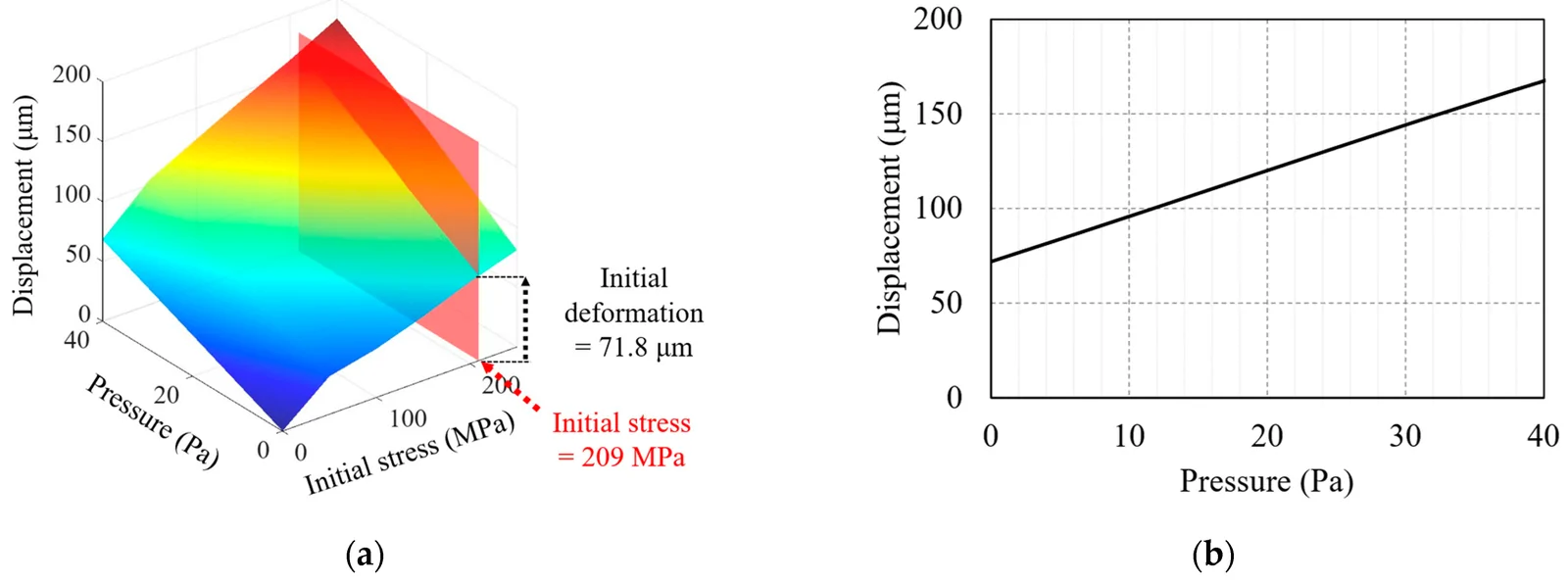
(**a**) Surface plot of pressure, initial stress and displacement with indication of measured initial deformation and (**b**) pressure to displacement curve with measured residual stress.

Figure 15a indicates the location corresponding to the measured initial deformation on the 3D surface relating the diaphragm initial stress, applied pressure, and cantilever-tip displacement. The residual stress corresponding to the measured initial deformation was estimated to be 209 MPa. The pressure–displacement curve with this stress applied is presented in Figure 15b From this plot, the stiffness was derived to be 0.16 N/m. The characteristic parameters of the diaphragm obtained through post-fabrication characterization are listed in Table 8.

**Figure 16:**
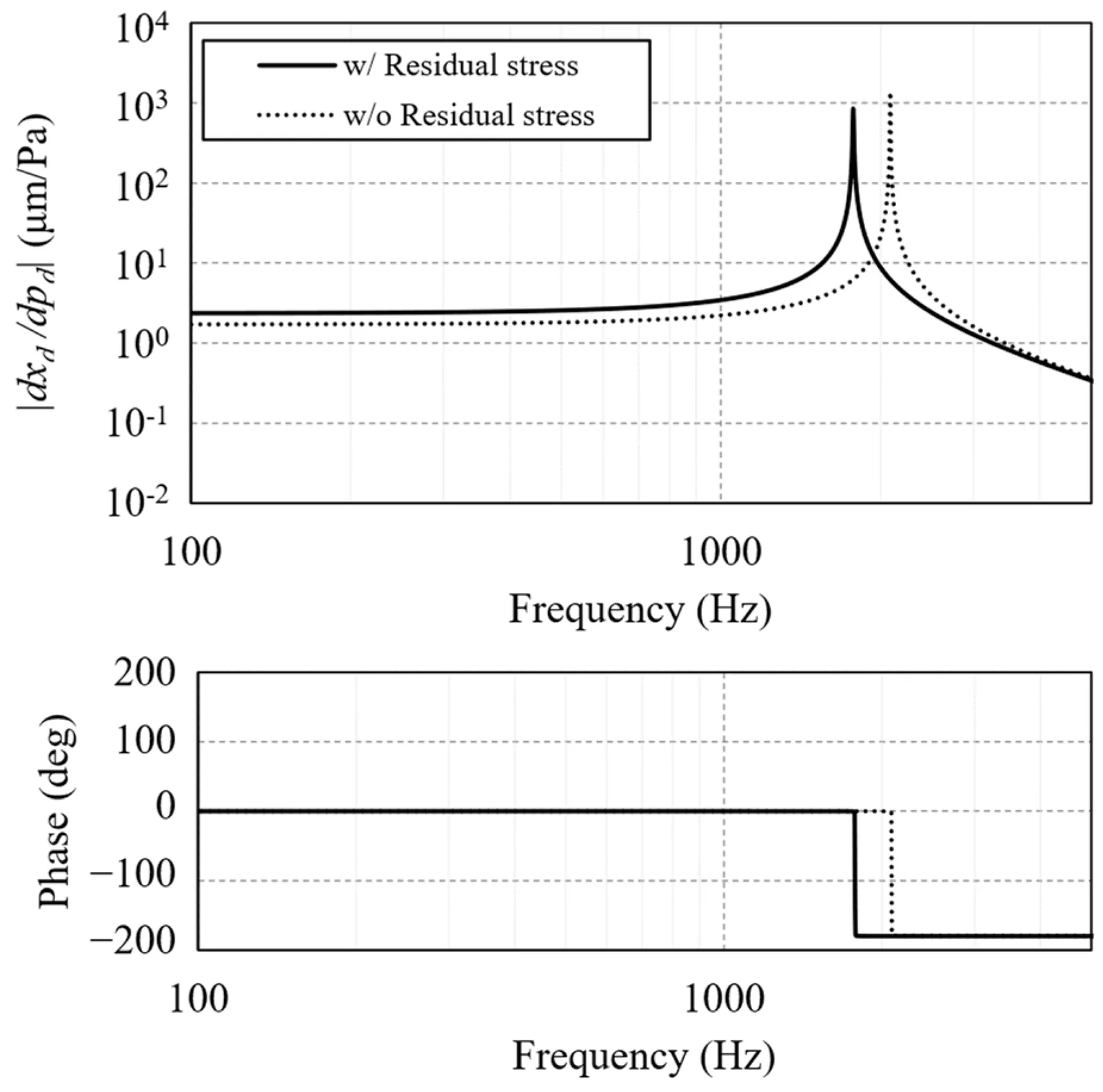
Comparison of the magnitude and phase frequency response of displacement by diaphragm incident pressure with and without residual stress.

Figure 16 presents the frequency response of the diaphragm displacement to the diaphragm incident pressure (*dx_d_*/*dp_d_*), before and after accounting for the residual stress. As a result of the reduced stiffness caused by the residual stress, the diaphragm resonance was calculated to decrease from the previously evaluated 2.09 kHz to 1.78 kHz.

**Figure 17:**
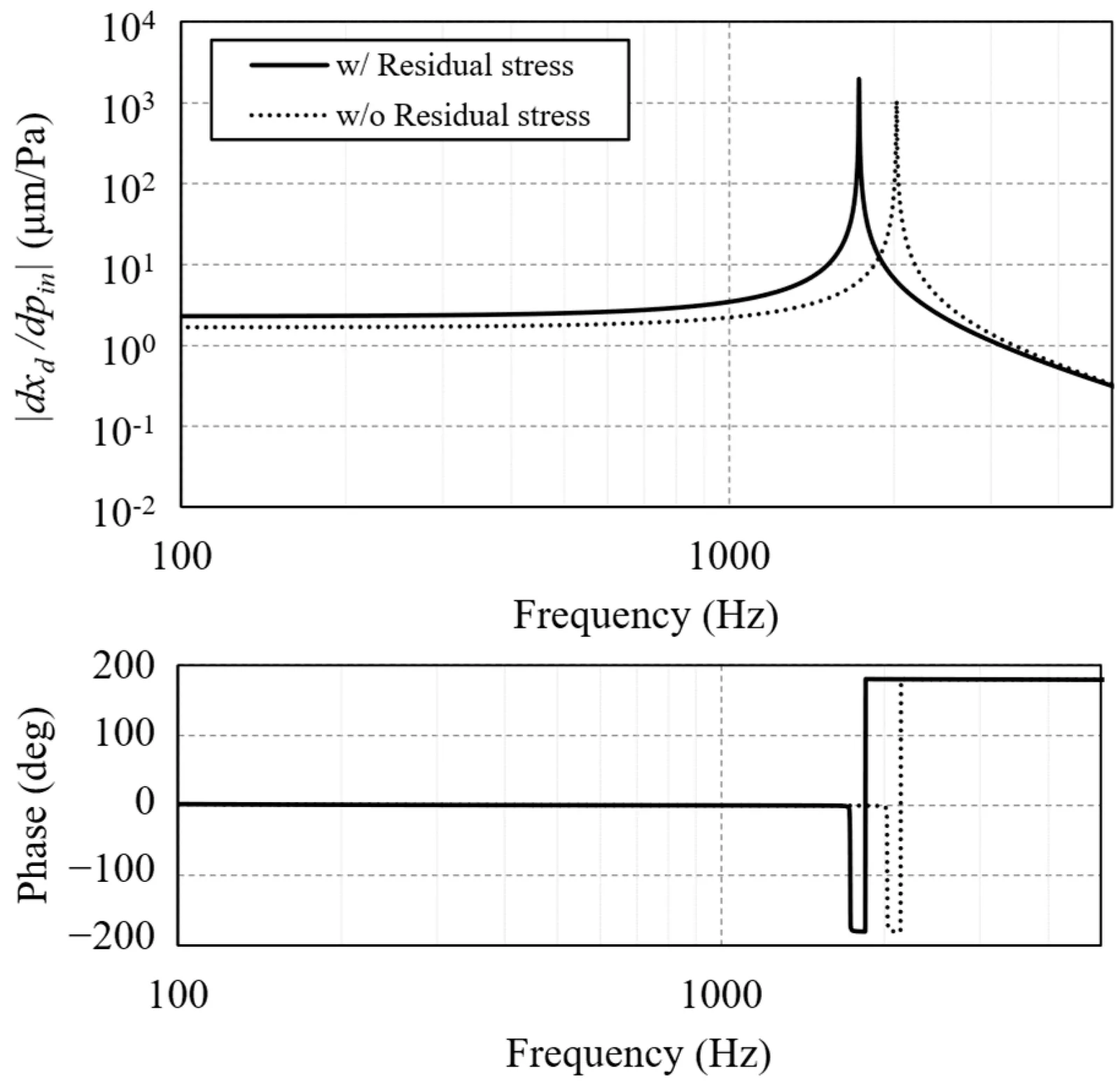
Comparison of magnitude and phase frequency response of displacement by sensor input pressure with and without residual stress.

Figure 17 presents the frequency response of the diaphragm displacement to the sensor input pressure, *dx_d_*/*dp_in_*, for the system incorporating the package acoustic network. After accounting for the residual stress, the resonance was observed at 1.73 kHz. As observed before accounting for the residual stress, the resonance frequency of the system incorporating the package was slightly lower than the intrinsic resonance frequency of the diaphragm. Consequently, 1.73 kHz was determined to be the design resonance frequency of the PrFET acoustic sensor with the cantilever diaphragm.

## 4. Experiment and Result

This section presents the experimental methods and results for the designed and fabricated PrFET acoustic sensor. To experimentally evaluate the response characteristics of the PrFET acoustic sensor, the fabricated sensor chip was integrated into the package case. An I–V converter was employed for conditioning the current signal. The acoustic response was then measured in an acoustic chamber and a semi-anechoic environment.

### 4.1. Experiment Setup

#### 4.1.1. Packaging

**Figure 18:**
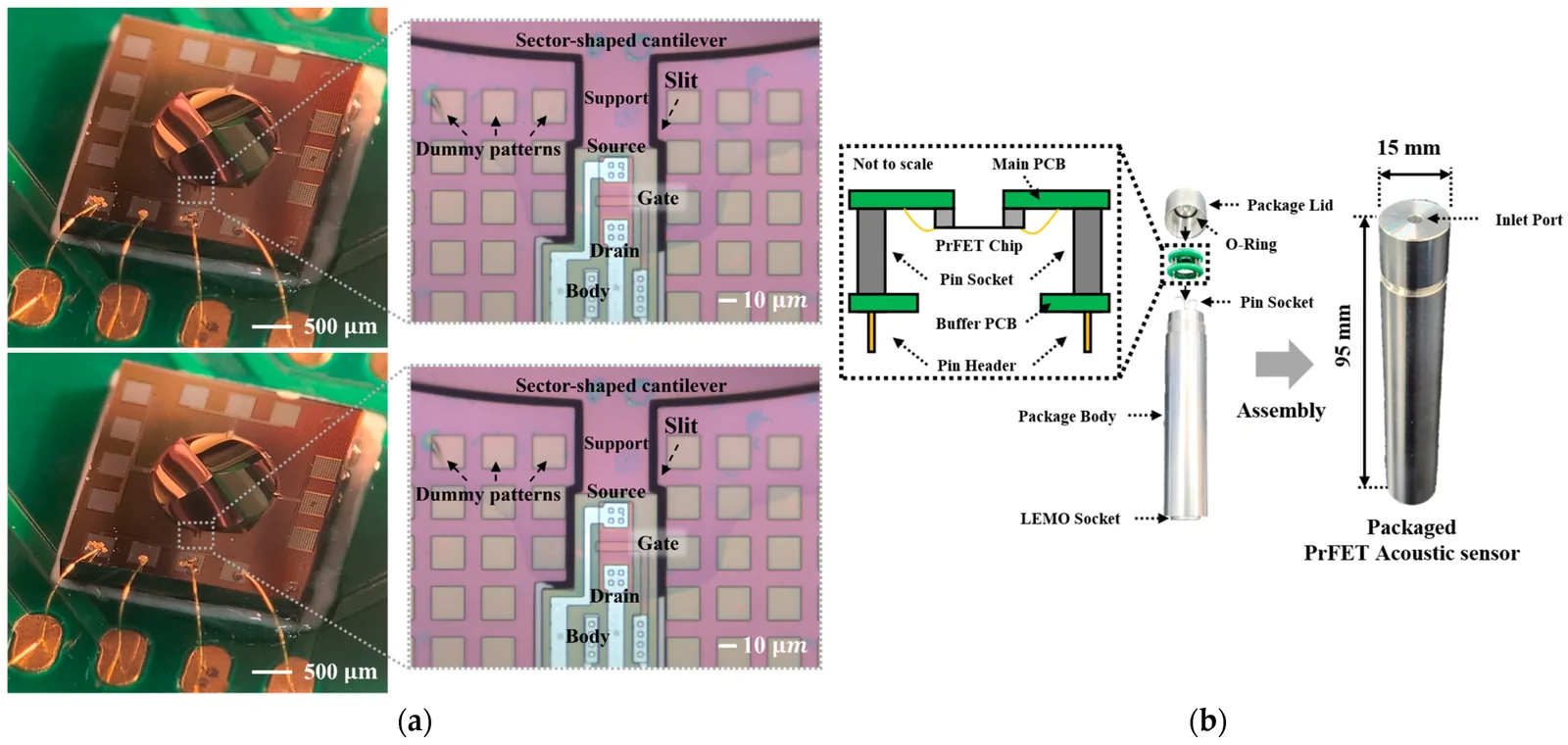
(**a**) The PrFET sensor chip (sector-shaped cantilever) wire bonded to the main PCB and (**b**) schematics of the PrFET cantilever chip bonded to the main PCB, and the packaged PrFET cantilever.

Figure 18a shows a single chip with a cantilever diaphragm obtained via dicing, wire-bonded onto a printed circuit board (PCB). The unused MOSFETs both ensured mechanical symmetry and served as backups. A 3 × 3 mm chip was attached to the main PCB using epoxy. The 1.2 mm-thick PCB featured a central acoustic port with a diameter of 1.7 mm and signal pads that could be wire-bonded to the cantilever chip. The main PCB was connected to a buffer PCB. The pin sockets protected the cantilever chip. Then, the PCB was assembled into an aluminum package, as shown in Figure 18b. In the package lid and body, rubber O-rings were inserted at the contact areas with the PCB in order to relieve packaging stress. The acoustic characteristics of the package that affect the pressure incident on the diaphragm were previously presented in Figure 9 of Section 2.2.3. The package is the same as that used in the previous PrFET acoustic sensor employing the circular diaphragm. Therefore, the package-related acoustic characteristics are maintained [24].

#### 4.1.2. I-V Converter

**Figure 19:**
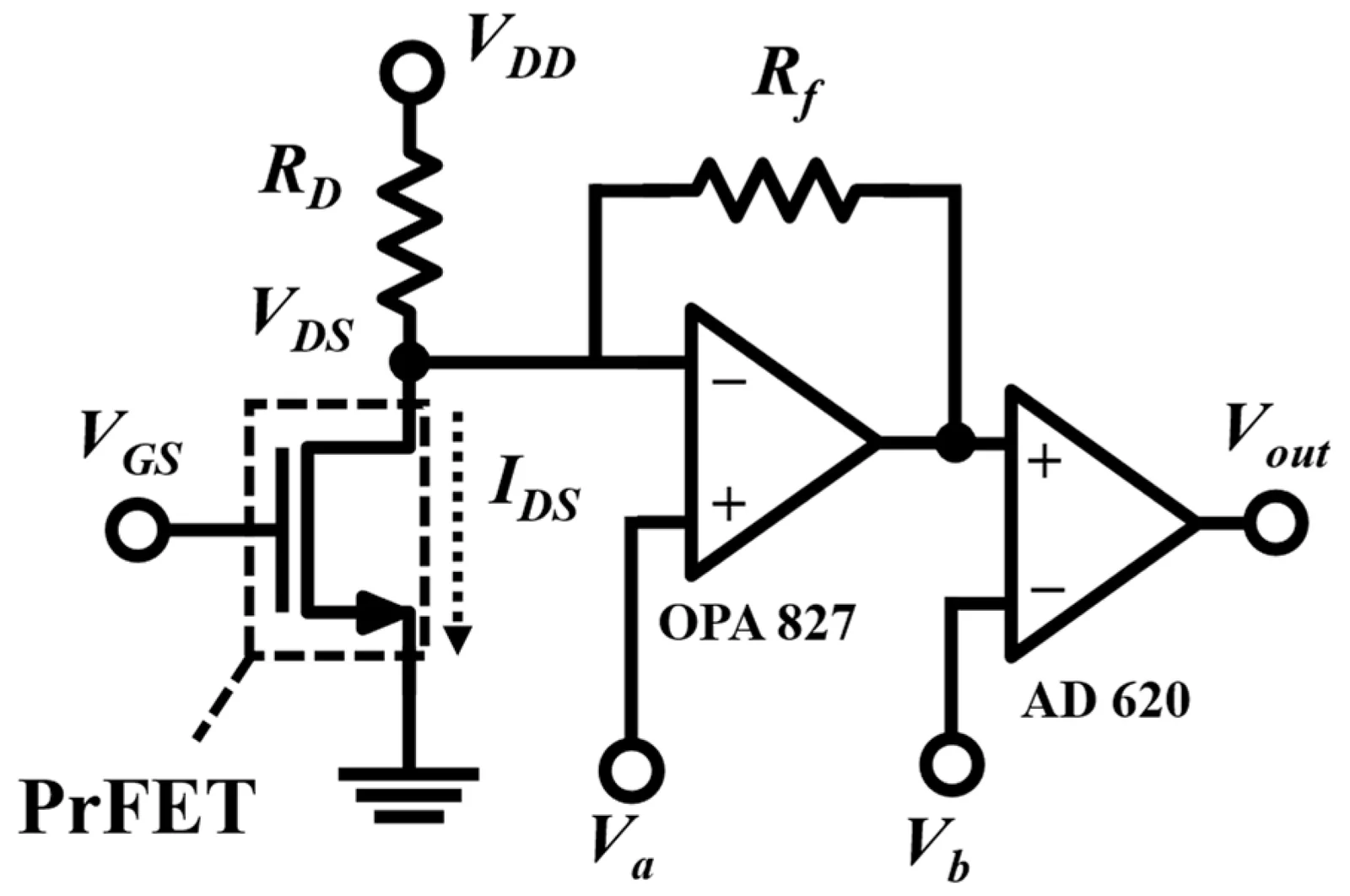
The read-out schema of the PrFET acoustic sensor when a transimpedance amplifier (an I-V converter) is employed.

The signal response of the source-drain current (*I_DS_*) change was measured by converting it into a voltage signal using an I-V converter (also termed a transimpedance amplifier). Figure 19 presents the I-V converter, which features two stages of operational amplifiers (op-amps). In the first stage (OPA827), the MOSFET operating point was determined by both the gate voltage (*V_GS_*) and the voltage at the non-inverting input (*V_a_*), because the drain node was connected to the inverting input of the op-amp (*V_DS_* = *V_a_*). *V_a_* was set to *V_GS_* to allow operation in the saturation region. The gain of the first stage, *R_f_*, was 6.8 kΩ. In the second stage (AD620), the DC bias component was removed using the *V_b_* of the op-amp. The AC component of the first-stage output then became the final output voltage (*V_out_*). This I–V converter was intended to receive the sensing diaphragm’s mechanical response without filtering. When the current signal in Equation (3) is considered in the context of the I-V converter and sensor input pressure, *p_in_*, the sensitivity of the output voltage to the input acoustic pressure (*dV_out_*/*dP_in_*) is given as follows:

(18) $$\frac{dV_{\mathrm{out}}}{dp_{\mathrm{in}}}=R_{f}\times \frac{dI_{\mathrm{DS}}}{dp_{\mathrm{in}}}$$

#### 4.1.3. Acoustic Measurement Setup

**Figure 20:**
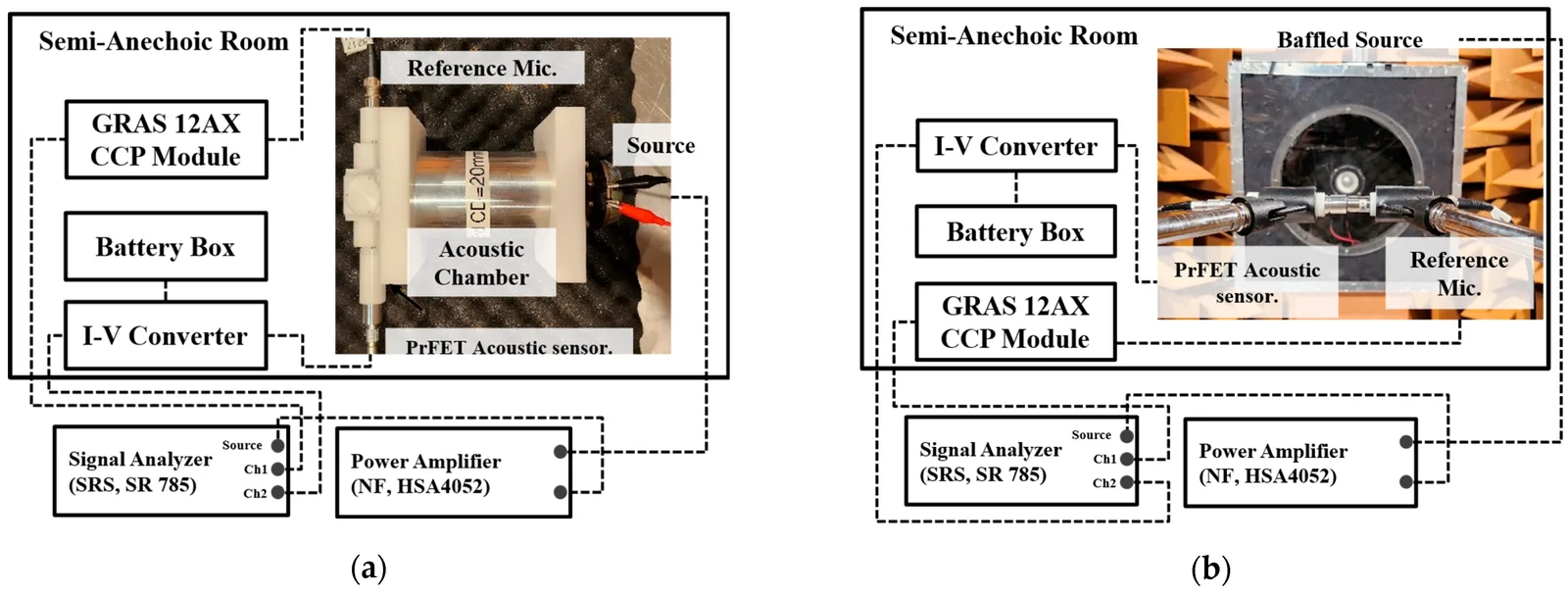
Experimental acoustic measurements in the (**a**) low-frequency and (**b**) high-frequency regions.

The sensitivities at various frequencies were measured in an acoustic chamber and a semi-anechoic room. In the low-frequency range, the acoustic output from the source may not be sufficiently delivered to the sensor under test in an open environment [23]. Therefore, low-frequency calibration below 1 kHz was performed in an enclosed acoustic chamber containing the speaker as the acoustic source, the PrFET acoustic sensor, and a reference microphone (Figure 20a). In contrast, at higher frequencies, higher-order acoustic modes can develop inside the chamber, causing the reference microphone and the device under test to be exposed to different sound pressure levels. Therefore, high-frequency calibration above 1 kHz was performed in a semi-anechoic environment instead of in the acoustic chamber, placing the reference microphone and the PrFET acoustic sensor at the same position relative to a baffled source (Figure 20b). In both cases, the sensors were calibrated against a reference microphone (46AO; G.R.A.S) using a signal analyzer (SR785; Stanford Research) in a semi-anechoic room with dimensions of 3 × 3 × 2 m.

### 4.2. Sensitivity Frequency Response

Using the setup described above, the sensitivity response of the PrFET acoustic sensor incorporating the designed cantilever diaphragm was measured. This sensitivity response is determined by the characteristics of the diaphragm, MOSFET, and package, as described by Equation (18). Therefore, the expected sensitivity response was calculated based on the previously characterized properties of the diaphragm, MOSFET, and package. The gate voltage was varied from 2 V to 5 V, and the source-drain voltage was set equal to the gate voltage to ensure operation in the saturation region.

**Figure 21:**
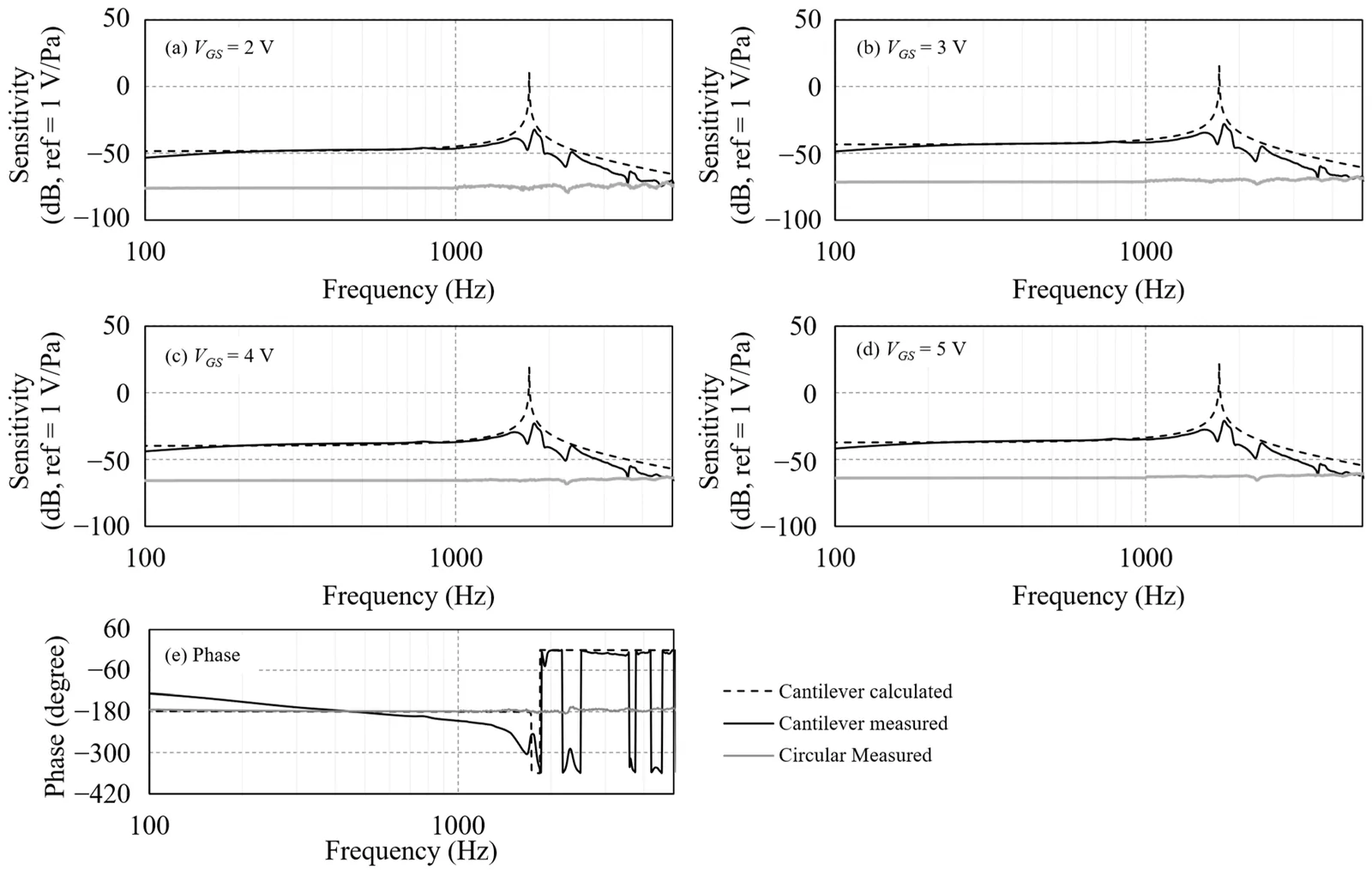
The calculated and measured sensitivity frequency responses of the PrFET acoustic sensors at the gate voltage (**a**) 2 V, (**b**) 3 V, (**c**) 4 V, (**d**) 5 V and (**e**) phase response.

Figure 21 presents the calculated and measured responses of the PrFET sensor with the cantilever diaphragm (Cantilever device), together with the measured response of the previously reported PrFET acoustic sensor with the circular diaphragm (Circular device) [17]. For all gate voltages, the measured sensitivity response of the cantilever device exhibited a resonance at 1.79 kHz and a relatively flat response from 200 Hz to frequencies below the resonance. The calculated response exhibited a resonance at 1.73 kHz, which was consistent with that predicted from the displacement response to the sensor input pressure presented in Figure 17. The measured resonance frequency was approximately 3.3% higher than the calculated value. These results demonstrate that the design approach adopted in this study can predict the resonance frequency of a PrFET acoustic sensor incorporating the designed sector-shaped cantilever diaphragm.

In contrast, the circular device exhibited a relatively flat response over the measured frequency range. In the flat-response region, the measured sensitivity of the cantilever device was approximately 30 dB higher than that of the circular device. Since the cantilever and circular devices had identical MOSFET and package characteristics, except for the diaphragm structure, the sensitivity enhancement and resonance observed in the cantilever device can be attributed to the structural characteristics of the diaphragm.

### 4.3. Linearity

**Figure 22:**
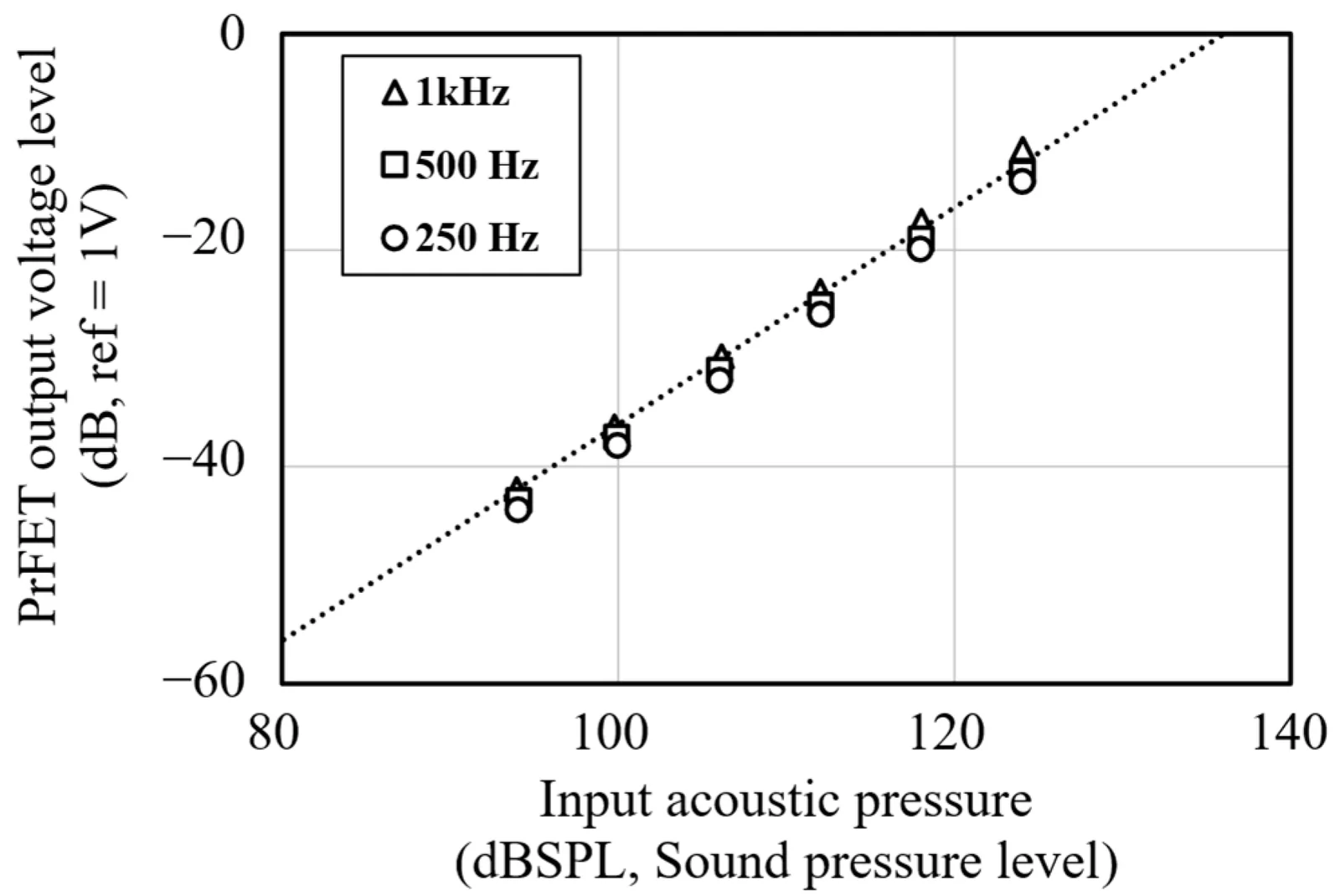
The linear relationship between the applied acoustic pressure and the output voltage of the cantilever diaphragm device.

Figure 22 shows the relationship between the applied acoustic pressure level and the output voltage level at a gate voltage of 3 V. The experimental setup was identical to that employed to assess sensitivity. The acoustic input pressure ranged from 94 to 124 dB SPL (1~32 Pa). To explore linearity, the cantilever device’s output voltages were measured when the source was driven at different frequencies lower than the resonance: 250 Hz, 500 Hz, and 1 kHz. Unlike resonance, where large diaphragm deformation may introduce nonlinearities, operation within this flat-response range is more suitable for applications requiring a linear response. The measurement results show that the cantilever device responded linearly to the applied acoustic pressure up to 124 dB SPL.

## 5. Discussion

Although the measured and calculated responses of the cantilever device exhibited agreement in the resonance frequency and flat response level below the resonance, discrepancies also existed between them. First, more than one resonance peak was observed in the measured response. The peak with the highest level is located at 1.79 kHz, but additional sensitivity peaks are observed at 1.58 kHz and 2.37 kHz. This may have been caused by unintended resonance behavior of the sensor system incorporating the diaphragm, the sensor die or the package. As resonant behaviors other than the diaphragm’s first mode were not considered in the design, they may contribute to the unintended resonance peaks observed in the experimental results. As shown in Supplementary Figure S1, the second resonance mode of the sector-shaped cantilever diaphragm occurred at 4.28 kHz, which may have contributed to the observed additional peak. Alternatively, it may be attributed to the assembly or mounting conditions of the sensor chip and PCB within the package, or to the characteristics of the semi-anechoic chamber in which the measurements were performed. Another possible explanation is acoustic or mechanical coupling between cantilevers with different resonance frequencies. As shown in Figure 14, the initial deformation of the four separated cantilevers is not uniform, so the resonance characteristics of each cantilever may also differ. When arrays of micromechanical cantilevers have different resonance characteristics from one another, additional resonances and asymmetric interference peaks may arise in their individual vibration responses due to mechanical or acoustic coupling [51]. To validate the coupling phenomena and their effects on the response, it is necessary to characterize the properties of each cantilever individually and evaluate the influence of mechanical and acoustic coupling between the cantilevers.

Furthermore, reductions in the response were observed immediately before each resonance peak. Consequently, although the measured and calculated responses showed good agreement in the flat response range below resonance, discrepancies were observed in the shape and level of the resonance peaks. In the measured response, a reduction in amplitude was observed at 1.69 kHz, immediately before the dominant resonance peak at 1.79 kHz. This reduction may also be attributed to coupling between multiple cantilevers. The validity of the damping characterization in the design model should be evaluated by comparison with the measured resonance peak level and bandwidth. However, in the absence of the response reduction at 1.69 kHz, which is presumed to arise from inter-cantilever coupling, the resonance peak level could be higher. Therefore, experimental validation of the damping characteristics of an individual cantilever would require either eliminating or characterizing the influence of coupling behavior on the response.

Another discrepancy was the low-frequency cutoff observed at 200 Hz in the measured response. The calculated response remained flat below 200 Hz. This discrepancy was presumed to result from the acoustic resistance of the vent slit. As shown in Figure 14, the vent slit of the cantilever diaphragm was enlarged from its designed dimensions due to deformation caused by residual stress. The front and back chambers are connected through the vent slit, causing the pressures in the two chambers to approach equilibrium at low frequencies [52]. As a result, the acoustic pressure incident on the diaphragm, *p_d_*, can decrease. Enlargement of the vent slit further reduces its acoustic resistance, causing the reduction in pd to extend to higher frequencies. The cutoff frequency, defined as the frequency at which the sensitivity response is 3 dB lower than the reference response level, was assumed to be determined by the acoustic resistance of the vent slit and the acoustic compliance of the back chamber as follows:

(19) $$f_{\mathrm{cutoff}}=\frac{1}{2\pi R_{a,s}C_{a,\mathrm{BC}}}$$

**Figure 23:**
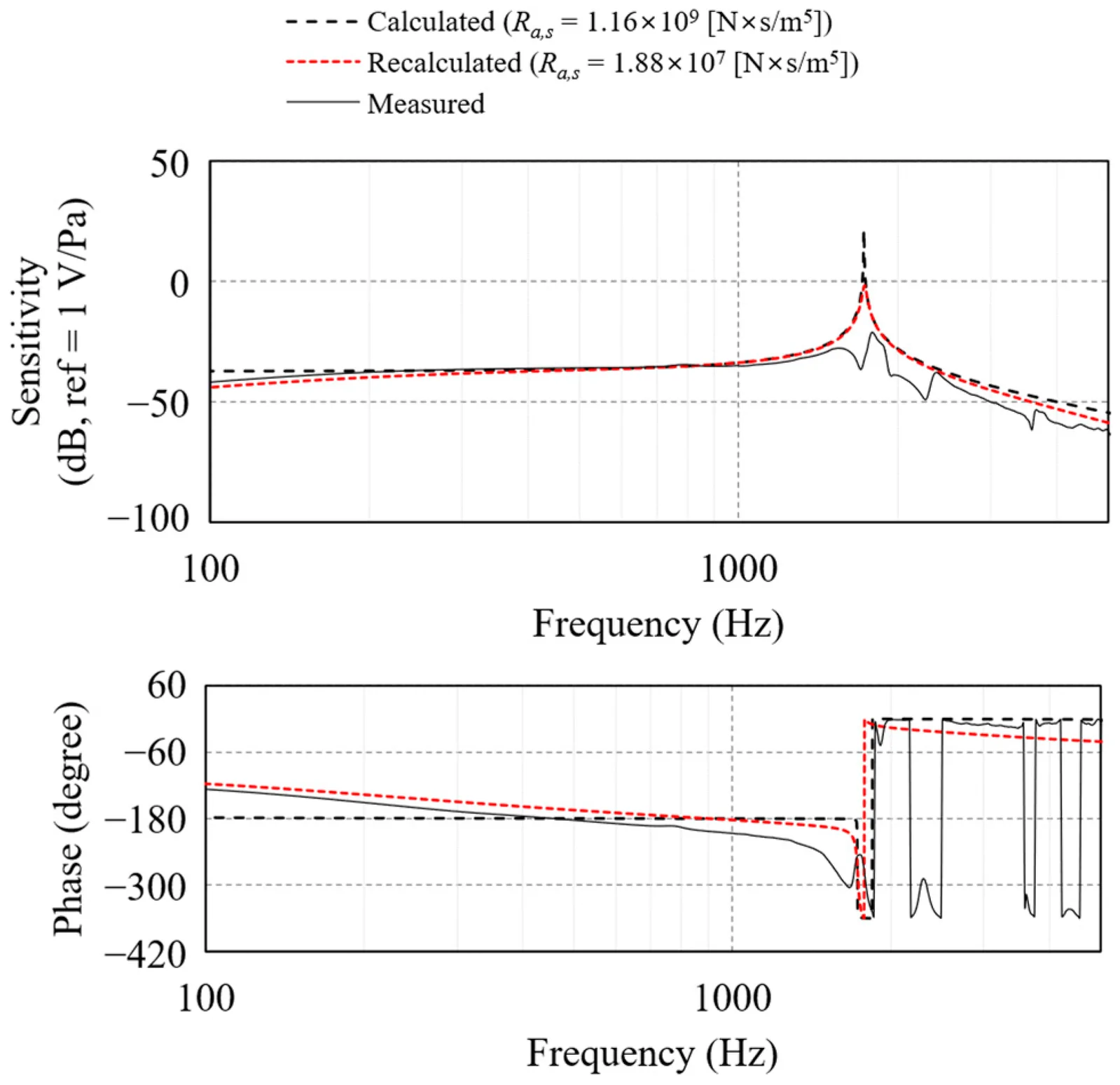
Sensitivity magnitude and phase response obtained using the value of vent-slit acoustic resistance in former and re-calculation.

The acoustic resistance of the vent slit was originally calculated without consideration of initial deformation. The recalculated vent slit resistance from the measured cutoff frequency is *R_a_*_,*s*_ = 1.88 × 10^7^ [N × s/m^5^]. Figure 23 compares the responses obtained using the former resistance value (*R_a_*_,*s*_ = 1.16 × 10^9^ [N × s/m^5^]) and the recalculated value (*R_a_*_,*s*_ = 1.88 × 10^7^ [N × s/m^5^]). The recalculated resistance was substantially lower than the former value, decreasing to approximately 1.6% of it. With this revised resistance, the calculated response showed better agreement with the measured response reduction at 200 Hz. Therefore, the model suggests that the enlargement of the vent slit reduced its acoustic resistance, resulting in a shift in the low-frequency cutoff to a higher frequency. However, the acoustic resistance was only recalculated from the observed low-frequency cutoff, and further characterization is required through measurement of the actual geometry of the deformed vent slit. To maintain the designed geometry of the vent slit, meanwhile, appropriate fabrication strategies will be required. The current enlargement of the vent slit is attributed to excessive tensile residual stress in the deposited silicon dioxide layer. Therefore, this tensile stress may need to be compensated through fabrication processes such as ion implantation or the deposition of an additional passivation layer with compressive residual stress [53,54].

## 6. Conclusions

As a result of employing the proposed sector-shaped cantilever diaphragm, a resonance was observed at 1.79 kHz, and the sensitivity was enhanced in the flat-response range below resonance. Because the MOSFET and package characteristics remained consistent with those of the previous device, these results demonstrate that the sector-shaped cantilever’s mechanical characteristics enable enhanced sensitivity within a specific frequency range. Furthermore, the approach adopted in this study was shown to be capable of designing the sensor resonance frequency with the proposed sector-shaped cantilever diaphragm. The results of this study may provide a basis for the development of various PrFET acoustic sensors for monitoring acoustic signals at a specific frequency.

Some discrepancies were observed between the calculated and measured responses. For the accurate design of the sensor response characteristics, the loss mechanisms other than thermoelastic damping should be investigated. Next, the acoustic resistance of the vent slit deformed by residual stress needs to be accurately characterized. It was only recalculated based on the observed low-frequency cutoff, so characterization based on accurate measurements of the actual vent slit geometry should be conducted. In addition, the unintended resonance behavior within the sensor system and potential coupling phenomena between multiple cantilevers should also be verified regarding the multiple resonance peaks. Eliminating or characterizing these coupling effects would enable validation of the damping characterization in this study. Furthermore, the noise characteristics of the mechanical–acoustic structure were not investigated. The I–V converter’s noise characteristics were also not evaluated, as it was employed to receive the mechanical response of the sector-shaped cantilever diaphragm without filtering. However, for practical application, noise characterization should be conducted, and appropriate improvement strategies should be developed. Meanwhile, further studies will also be conducted to broaden and improve the performance characteristics of the PrFET acoustic sensor. These studies may include the application of more diverse sensing structures, the investigation of acoustic structures and MOSFET designs, and the development of signal-conditioning schemes, such as those using multiple MOSFETs.

## Figures and Tables

**Table 1:** Recently reported studies on piezoresistive MEMS acoustic sensors including their sensing element, diaphragm areas, resonance frequencies, sensitivities, acoustic overload point.

Work	Sensing Element	Diaphragm Area [mm^2^]	*f_r_* [kHz]	Sensitivity (Ref 1 V/Pa)	Acoustic Overload Pressure
Zhou et al. (2012) [25]	Metal-induced lateral crystal Poly-Si (p-type) piezoresistor	1.32 × 10^−8^	400	−122 dB *	N/R
Lee et al. (2019) [26]	Silicon nanowire piezoresistor	1.56	N/R	N/R	N/R
Wei et al. (2026) [27]	Graphene piezoresistors	0.25	64.07	−119.07 dB **	170 dBSPL **
Kim et al. (2024) [24]	PrFET (Piezoresistive channel MOSFET)	1.54	10	−63.79 dB ***	134 dBSPL ***

* Measured using a reference microphone under spark-generated shock wave excitation. ** Predicted based on design and simulation, not experimental measurement results. *** Measurements were conducted in a sealed chamber coupled to a loudspeaker below 1 kHz and in a semi-anechoic chamber above 1 kHz.

**Table 2:** The geometric dimensions commonly shared by the circular and the cantilever diaphragm.

Parameter	Description	Value	Units
*t_c_*	Diaphragm thickness	2.68	μm
*r_s_*	Diaphragm radius	700	
*w_slit_*	Slit width	4	
*l_s_*_1_	Length of support 1	55	
*w_s_*_1_	Width of support 1	40	
*l_s_*_2_	Length of support 2	62	
*w_s_*_2_	Width of support 2	50	

**Table 3:** Material properties of the constituent materials of the cantilever diaphragm related to damping characteristics.

Parameter	Description	Value		Unit
		Silicon	Silicon Dioxide	
*E*	Young’s modulus	169 [44]	70 [45]	GPa
*χ*	Thermal diffusivity	88 [46]	0.83 [47]	mm^2^/s
α	Thermal expansion coefficient	2.6 × 10^−6^ [48]	0.5 × 10^−6^ [45]	1/K
*C_P_*	Specific heat capacity	700 [48]	710 [49]	J/(kg × K)

**Table 4:** Cantilever diaphragm characteristic parameters associated with displacement response.

Parameter	Description	Central Angle (*θ_c_*)					Unit
		30°	45°	60°	75°	90°	
*A_d_*	Sensing area	0.13	0.20	0.26	0.32	0.39	×10^−6^ m^2^
*k_d_*	Stiffness	0.25	0.25	0.24	0.23	0.23	N/m
*m_d_*	Mass	0.40	0.63	0.84	1.04	1.32	×10^−9^ kg
*R_m_*_,*d*_	Damping coefficient	0.94	1.18	1.33	1.45	1.64	×10^−9^ N × s/m

**Table 5:** Symbols and expressions of acoustic impedance of each structure.

Structure	Impedance Symbol	Expression
Diaphragm	*Z_d_*	$\mathrm{j\omega}\frac{m_{d}}{{A_{d}}^{2}}+\frac{k_{d}}{\mathrm{j\omega}{A_{d}}^{2}}+\frac{R_{m,d}}{{A_{d}}^{2}}$
Inlet hole	*Z_i_*	*jωL_a_*_,*i*_ + *R_a_*_,*i*_
Vent slit	*Z_s_*	*jωL_a_*_,*s*_ + *R_a_*_,*s*_
Front chamber	*Z_FC_*	$\frac{1}{\mathrm{j\omega}C_{a,\mathrm{FC}}}$
Back chamber	*Z_BC_*	$\frac{1}{\mathrm{j\omega}C_{a,\mathrm{BC}}}$

**Table 6:** Values of acoustic impedance components.

Parameter	Description	Value	Unit
*L_a_*_,*i*_	Acoustic inertance of the inlet port	7.32 × 10^2^	kg/m^4^
*R_a_*_,*i*_	Acoustic resistance of the inlet port	2.79 × 10^4^	N × s/m^5^
*C_a_*_,*FC*_	Acoustic compliance of the front chamber	2.61 × 10^−14^	N/m^5^
*C_a_*_,*BC*_	Acoustic compliance of the back chamber	4.24 × 10^−11^	
*L_a_*_,*s*_	Acoustic inertance of the slit	1.37 × 10^2^	kg/m^4^
*R_a_*_,*s*_	Acoustic resistance of the slit	1.16 × 10^9^	N × s/m^5^

**Table 7:** The measured MOSFET parameters.

Parameter	Description	Gate Voltage				Unit
		2 V	3 V	4 V	5 V	
*K_n_*	Transconductance parameter	1674	892	368	270	μA/V^2^
*V_t_*	Threshold voltage	1.2				V

**Table 8:** Cantilever diaphragm characteristic parameters before and after accounting for residual stress.

Parameter	Description	Value		Unit
		w/o Residual Stress	w/ Residual Stress	
*A_d_*	Sensing area	0.39	0.39	×10^−6^ m^2^
*k_d_*	Stiffness	0.23	0.16	N/m
*m_d_*	Mass	1.32	1.32	×10^−9^ kg

## Data Availability

The data presented in this study are available on request from the corresponding author. The data are not publicly available because they are part of an ongoing study.

## Supplementary Materials

The following supporting information can be downloaded at https://www.mdpi.com/article/10.3390/s26196274/s1. Figure S1: Eigenfrequency result of the sector-shaped cantilever diaphragm’s second mode.
