# Supplementary material for: A Study on a Sector-Shaped Cantilever Diaphragm for Frequency-Response Improvement of an Acoustic Sensor Based on Piezoresistive Effect in MOSFET
Source: Sensors (Basel). 2026 Oct 3;26(19):6274. doi: 10.3390/s26196274 (PMC13667680; doi:10.3390/s26196274)
Supplement: Supplementary file 1 [file sensors-26-06274-s001.zip › sensors-4548396-supplementary.pdf]

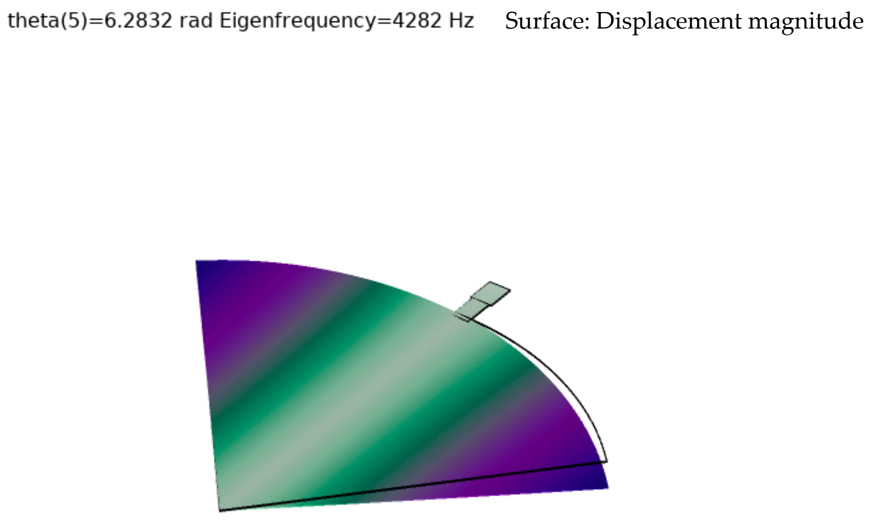

Figure S1. Eigenfrequency result of the sector-shaped cantilever diaphragm's second mode.
